# Synthesis of Optically and Redox Active Polyenaminones from Diamines and α,α’-Bis[(dimethylamino)methylidene]cyclohexanediones

**DOI:** 10.3390/polym14194120

**Published:** 2022-10-01

**Authors:** Urša Štanfel, Tomaž Kotnik, Sebastijan Ričko, Uroš Grošelj, Bogdan Štefane, Klemen Pirnat, Ema Žagar, Boštjan Genorio, Jurij Svete

**Affiliations:** 1 Faculty of Chemistry and Chemical Technology, University of Ljubljana, Večna Pot 113, 1000 Ljubljana, Slovenia; 2 National Institute of Chemistry, Department of Materials Chemistry, Hajdrihova 19, 1000 Ljubljana, Slovenia; 3 National Institute of Chemistry, Department of Polymer Chemistry and Technology, Hajdrihova 19, 1000 Ljubljana, Slovenia

**Keywords:** polyenaminones, transaminative polymerisation, bis-enaminones, diamines, UV shielding, redox activity

## Abstract

New oligo- and polyenaminones with *M_w_* ~ 7–50 KDa were prepared in high yields by transaminative amino-enaminone polymerization of regioisomeric bis[(dimethylamino)methylidene]cyclohexanediones with alkylene and phenylenediamines. The polymers obtained are practically insoluble in aqueous and organic solvents and exhibit film-forming properties, UV light absorption at wavelengths below 500 nm, and redox activity. These properties indicate a promising application potential of these polymers, which could find use in optical and optoelectronic applications and in energy storage devices.

## 1. Introduction

In the past century, the development of novel synthetic polymeric materials have made an important contribution to modern technologies and society. Various diamines are standard bifunctional monomer building blocks used in the industrial production of polyamides and other aminopolymers, such as conductive polyimines [1,2,3,4]. In contrast to polyamides as multi-purpose plastic materials, the related polyenamines are little studied nowadays [5]. Polyenaminones are vinylogous polyamides accessible by polymerization between diamines and β-alkoxyenones [6,7,8,9,10,11,12], diketene [13,14], diynones [15,16,17], and bis(N,N-disubstituted) enaminones [18,19,20]. Due to the presence of a conjugated enaminone push-pull system, polyenaminones absorb UV and visible light. In addition, film-forming properties [19] and their degradability and recyclability under mild conditions, either by hydrolysis with strong acids [12,19] or by transamination with excess dimethylamine [20], have been recently demonstrated. The large number and variety of accessible starting materials, ease of preparation, tunable structure, and promising properties of polyenaminones warrant further investigation in this field.

**Figure 1:**
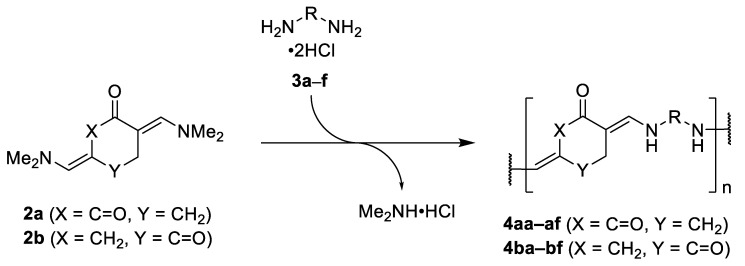
Preparation of polyenaminones **4** by acid-catalysed polymerisation of diamines **3a**–**f** with α,α-bis[(dimethylamino)methylidene]cyclohexanediones **2a**,**b**.

Continuing our work on polyenaminones [19,20], we have dealt with bis-enaminones **2a**,**b** derived from 1,2-cyclohexanedione (**1a**) and 1,4-cyclohexanedione (**1b**). Compounds **1a** and **1b** were chosen as starting materials because they are accessible from biomass-derived precursors [21,22,23,24,25,26] and because they are saturated analogues of the respective quinones and hydroquinones. The latter are electroactive organic molecules of great importance in various natural chemical and biochemical cycles (photosynthesis, aerobic respiration, etc.) [27]. Recently, electroactive organic molecules have attracted great interest in energy storage devices due to their large abundance, natural occurrence, and easy accessibility. Moreover, electroactive organic molecules can be easily functionalized and tuned in terms of redox potential. Redox-active molecules can be used as cathode materials in lithium or beyond-lithium battery systems. However, the solubility in most organic electrolytes is a major drawback because the capacity decreases rapidly. The problem of solubility can be solved by several approaches. One of these approaches is polymerization, which usually yields insoluble or poorly soluble polymer chains with electroactive moieties [28]. For example, the transaminative polymerization of 3,6-bis [3-(dimethylamino)methylidene]cyclohexane-1,2-dione (**2a**) and 2,5-bis [3-(dimethylamino)methylidene]cyclohexane-1,4-dione (**2b**) with diamines **3a**–**f** should lead to the formation of poorly soluble polyenaminones **4**, which could also be redox-active (Figure 1).

In the present study, we tested quinone precursors **4** that are not conjugated (lacking a double bond). However, because of their close similarity to *ortho*- and *para*-benzoquinones, we tested polyenaminones **4** as cathode materials in Li batteries for possible redox activity. We report here the results of this study, which confirmed the feasibility of the synthesis method and showed promising redox activity of polyenaminones **4**.

## 2. Experimental

### 2.1. Materials and General Methods

#### 2.1.1. Materials

All solvents and reagents were used as received. Cyclohexane-1,2-dione (**1a**, 97.0%), cyclohexane-1,4-dione (**1b**, 97.0%), *N*,*N*-dimethylformamide dimethylacetal (DMFDMA, for synthesis, 97.0%), 1,2-diaminoethane (**3a**) dihydrochloride (97.0%), 1,3-diaminopropane (**3b**) dihydrochloride (97.0%), 1,4-diaminobutane (**3c**) dihydrochloride (97.0%), *o*-phenylenediamine (**3d**) dihydrochloride (98.0%), *m*-phenylenediamine (**3e**) dihydrochloride (98.0%), and *p*-phenylenediamine (**3f**) dihydrochloride (99.0%) are commercially available (abcr GmbH, Karlsruhe, Germany).

#### 2.1.2. General Methods

Melting points (mp) were determined on a Kofler micro hot stage (Leica Galen III) (Leica Camera AG, Wetzlar, Germany).

The NMR spectra were recorded in DMSO-*d*_6_ as deuterated solvent using Me_4_Si as the internal standard on a Bruker Avance III UltraShield 500 plus instrument (Bruker, Billerica, MA, USA) at 500 MHz for ^1^H and at 126 MHz for ^13^C nucleus, respectively. Chemical shifts (δ) are given in ppm relative to Me_4_Si as internal standard (δ = 0 ppm) and vicinal coupling constants (*J*) are given in Hertz (Hz). The following common abbreviations are used for description of signals in ^1^H NMR spectra: singlet (s), broad singlet, (br s), and doublet (d). 

UV spectra were recorded in MeOH using a Varian Cary Bio50 UV-Visible Spectrophotometer (Varian, Palo Alto, CA, USA). The wavelengths of the absorption maxima (λ_max_) are given in nanometers (nm) and the extinction coefficients (ε) in dm^3^·mol^−1^·cm^−1^.

Fourier-transform infrared (FT-IR) spectra were obtained on a Bruker FTIR Alpha Platinum spectrophotometer (Bruker, Billerica, MA, USA) using attenuated total reflection (ATR) sampling technique. The absorption frequencies (ν_max_) in the IR spectra are given in cm^−1^. 

High resolution mass spectrometry (HRMS) analyses were obtained using **time-of-flight liquid chromatography/mass spectrometry** (**TOF LC/MS**). An Agilent 6224 time-of-flight (TOF) mass spectrometer equipped with a double orthogonal electrospray source at atmospheric pressure ionization (ESI) coupled to an Agilent 1260 high-performance liquid chromatograph (HPLC) (Agilent Technologies, Santa Clara, CA, USA) was used for recording HRMS spectra. Mobile phase composed of two solvents: A was 0.1% formic acid in Milli-Q water (Sigma-Aldrich, St. Louis, MO, USA), and B was 0.1% formic acid in acetonitrile mixed in the ratio of 1:1. Compounds were prepared by dissolving the samples in acetonitrile and 0.1–10 μL of each sample (*c* ~ 1 mg mL^−1^) was injected into the liquid chromatograph-mass spectrometer (LC-MS). Flow rate was 0.4 mL/min, fragmentor voltage was 150 V, capillary voltage 4000 V, and mass range was 100–1700. The following common abbreviations are used for description of HRMS data: mass-to-charge ratio (*m*/*z*) and protonated molecular ion (MH^+^).

Microanalyses for C, H, and N were obtained on a Perkin-Elmer CHN Analyzer 2400 II (PerkinElmer, Waltham, MA, USA). Stirring at room temperature was carried out on a Tehtnica Vibromix 313 EVT orbital shaker (Domel Holding, d.d., Železniki, Slovenia).

Size-exclusion chromatography (SEC) was performed on an Agilent Technologies 1260 Infinity series pump coupled to a VWD Agilent Technologies 1260 UV detector (λ = 280 nm) (Agilent Technologies, Santa Clara, CA, USA). Separation conditions: 40 °C, PL HFIPgel 9 μm column with a pre-column 300 mm × 7 mm (Agilent Technologies, Santa Clara, CA, USA), 0.01 M Et_4_N^+^NO_3_^–^ (TEAN) in (CF_3_)_2_CHOH (HFIP), flow = 1 mL min^−1^. SEC column was calibrated with poly(methyl methacrylate) (PMMA) standards of different, but defined molar masses and narrow molar mass distributions (Agilent Technologies, Santa Clara, CA, USA). Typical masses of the samples injected onto the column were 0.1 mg (*c* = 1.0 mg mL^−1^). Agilent GPC-Addon Rev.B01.01 (Agilent Technologies, Santa Clara, CA, USA) using a conventional calibration method was utilized for the data acquisition and evaluation.

Thermogravimetric (TG) measurements were performed on a Netzsch 449 F3 Jupiter instrument (Netzch, Selb, Germany) under a dynamic Ar (5.0) flow with a flow rate of 60 mL/min in a temperature range from 30 °C to 1200 °C. A heating rate of 10 K/min was used. About 10 mg of sample was placed in alumina (Al_2_O_3_) crucible. Simultaneously mass spectrometry was performed on MS 403C Aëolos mass spectrometer (Netzch, Selb, Germany) with a SEM Chenneltron detector (Photonis, Paris, France) and system pressure of 2 × 10^–5^ mbar. Gasses that evolved under TG heat treatment were transferred to the mass spectrometer through transfer capillary, quartz ID 75 μm (Sigma-Aldrich, St. Louis, MO, USA), which was heated up to 220 °C. The upper limit of the mass spectrometer detector was 100 AMU.

Polyenaminones microstructure characterization was performed by scanning field emission electron microscope Zeiss ULTRA plus (SEM) (Zeiss, Jena, Germany). Polyenaminones were adhered to the conductive carbon tape placed on aluminum SEM holder. Platinum-palladium, nominally 20 nm thick was evaporated on to the sample using Qurum Q150T ES turbomolecular pumped coater (AGC, Chraleroi, Belgium). SEM images were taken at 2 kV using SE2 detector at WD 4.5 mm.

Electrodes were prepared by mixing 60 mg of tested polymer material, 30 mg of carbon black (Printex XE2), and 10 mg of polytetrafluoroethylene (PTFE) (60 wt.% water dispersion) and 0.5 mL of isopropyl alcohol (IPA) (Sigma-Aldrich, St. Louis, MO, USA). All these ingredients were ball milled in 12 mL stainless steel grinding jars (10 mm Ø balls) with a planetary ball mill (Retsch PM100) at 300 rpm for 30 min in an air atmosphere (Retsch, Haan, Germany). The obtained slurry was kneaded with a mortar and pestle to obtain a compact black gum. The gum was rolled between two pieces of a nonadhesive paper with a roller to obtain an electrode film of approximately 5 cm × 5 cm size. An aluminum mesh (100 mesh size) was deposited on top of this film and the film was rolled again to glue the electrode composite and the mesh together and then dried in an air atmosphere. Afterward, electrode discs with a diameter of 1.2 cm were cut and pressed with a load of 1 ton and further dried at 60 °C in a vacuum for 1 day. The average loading on the electrode was around 2.5 mg of active material per cm^2^. Battery cells were assembled in an argon-filled glovebox (water and oxygen levels <1 ppm). Swagelok-type battery cells were assembled using the above-mentioned electrodes, a 13 mm glass fiber separator (Whatman GF/A) (Whatman plc, Maidstone, Kent, UK), and freshly rolled lithium (12 mm diameter) (Sigma-Aldrich, St. Louis, MO, USA). 1 M Bis(trifluoromethane)sulfonimide lithium salt in a mixture of dry 1,3-dioxolane/dimethoxyethane was used an electrolyte (1 M LiTFSI/DOL+DME) (Sigma-Aldrich, St. Louis, MO, USA). A potentiostat/galvanostat VMP3 (Bio-Logic, Seyssinet-Pariset, France) was used at room temperature (25 °C) to perform the electrochemical measurements. Batteries were galvanostatically cycled between 1.5–3.5 V vs. Li/Li^+^ at a current density of 50 mA/g according to mass of tested polymer.

### 2.2. General Procedure for the Synthesis of Compounds ***2a*** and ***2b***

A mixture of cyclohexanedione **1a**,**b** (1.121 g, 10 mmol), DMFDMA (3.0 mL, 22 mmol), and anhydrous PhMe (10 mL) was heated under reflux for 5 h. The reaction mixture was cooled to 20 °C, the precipitate was collected by filtration, and washed with toluene (2 × 5 mL) to give **2a**,**b**. The following compounds were prepared in this manner:

#### 2.2.1. (3*E*,6*E*)-3,6-bis[(dimethylamino)methylidene]cyclohexane-1,2-dione (**2a**)

Prepared from **1a** (1.121 g, 10 mmol). Light orange-brown solid (1.236 g, 56%); mp = 191–195 °C. FT-IR (ATR): ν_max_ 2947, 2837, 1644 (C=O), 1542, 1483, 1461 cm^−1^. ^1^H NMR (500 MHz; DMSO-d_6_): δ 2.64 (4H, s), 3.08 (12H, s), 7.41 (2H, s). ^13^C NMR (126 MHz; DMSO-d_6_): δ 23.9, 43.3, 105.3, 151.0, 184.1. (Found: C, 64.75; H8.33; N, 12.25. C_12_H_18_N_2_O_2_ requires C, 64.84; H, 8.16; N, 12.60%). *m*/*z* (HRMS): 223.1442 (MH^+^). C_12_H_19_N_2_O_2_ requires *m*/*z* 223.1441.

#### 2.2.2. (2*Z*,5*E*)-2,5-bis((dimethylamino)methylidene)cyclohexane-1,4-dione (**2b**)

Prepared from **1b** (1.121 g, 10 mmol). Black solid (1.082 g, 49%); mp = 131–135 °C. FT-IR (ATR): ν_max_ 2952, 2901, 2803, 1648 (C=O), 1546, 1423, 1291, 1156, 1090, 1067, 1001, 952, 870, 806, 731, 651 cm^−1^. ^1^H NMR (500 MHz; DMSO-*d*_6_): *δ* 2.16 and 2.44 (4H, 2d, 1:1, *J* = 11.0 Hz), 2.86 (12H, br s), 7.27 (2H, s). ^13^C NMR (126 MHz; DMSO-*d*_6_): *δ* 36.0, 39.6, 100.8, 145.4, 197.5. *m/z* (HRMS): 223.1442 (MH^+^). C_12_H_19_N_2_O_2_ requires *m/z* 223.1441.

### 2.3. General Procedure for the Synthesis of Compounds ***4aa*–*4af*** and ***4ba*–*4bf***

A mixture of compound **2** (222 mg, 1 mmol), diamine dihydrochloride **3** (1 mmol), and methanol (10 mL) was stirred at 20 °C for 72 h. The precipitate was collected by filtration, and washed with MeOH (2 mL) to give **4**. The following compounds were prepared in this manner:

#### 2.3.1. Poly{(*E*)-[3-({[2-(λ^2^-azaneyl)ethyl]amino}methylene)-6-(λ^3^-methylene)cyclohexane-1,2-dione]} (**4aa**)

Prepared from **2a** (222 mg, 1 mmol) and 1,2-ethylenediamine dihydrochloride **3a** (133 mg, 1 mmol). Yield: 180 mg (87%) of brown solid. FT-IR (ATR): ν_max_ 3650, 2917, 2225, 2017, 1630 (C=O), 1551 cm^−1^. λ_max_ (MeOH)/nm 342 (ε/dm^3^·mol^−1^·cm^−1^ 1318) and 297 (1545).

#### 2.3.2. Poly{(*E*)-[3-({[3-(λ^2^-azaneyl)propyl]amino}methylene)-6-(λ^3^-methylene)cyclohexane-1,2-dione]} (**4ab**)

Prepared from **2a** (222 mg, 1 mmol) and 1,3-propylenediamine dihydrochloride **3b** (146 mg, 1 mmol). Yield: 21 mg (10%) of brown-red solid. FT-IR (ATR): ν_max_ 2914, 2835, 1627 (C=O), 1505, 1436 cm^−1^. λ_max_ (MeOH)/nm 430 (ε/dm^3^·mol^−1^·cm^−1^ 9963).

#### 2.3.3. Poly{(*E*)-[3-({[4-(λ^2^-azaneyl)butyl]amino}methylene)-6-(λ^3^-methylene)cyclohexane-1,2-dione]} (**4ac**)

Prepared from **2a** (222 mg, 1 mmol) and 1,3-butylenediamine dihydrochloride **3c** (161 mg, 1 mmol). Yield: 31 mg (13%) of purple-red solid. FT-IR (ATR): ν_max_ 3222, 2931, 2832, 1628 (C=O), 1517, 1439 cm^−1^. λ_max_ (MeOH)/nm 433 (ε/dm^3^·mol^−1^·cm^−1^ 9870).

#### 2.3.4. Poly{(*E*)-[3-({[2-(λ^2^-azaneyl)phenyl]amino}methylene)-6-(λ^3^-methylene)cyclohexane-1,2-dione]} (**4ad**)

Prepared from **2a** (222 mg, 1 mmol) and 1,2-phenylenediamine dihydrochloride **3d** (181 mg, 1 mmol). Yield: 122 mg (48%) of brown solid. FT-IR (ATR): ν_max_ 2831, 2116, 1999, 1628 (C=O), 1595, 1499 cm^−1^. λ_max_ (MeOH)/nm 446 (ε/dm^3^·mol^−1^·cm^−1^ 3755) and 372 (3303).

#### 2.3.5. Poly{(*E*)-[3-({[3-(λ^2^-azaneyl)phenyl]amino}methylene)-6-(λ^3^-methylene)cyclohexane-1,2-dione]} (**4ae**)

Prepared from **2a** (222 mg, 1 mmol) and 1,3-phenylenediamine dihydrochloride **3e** (181 mg, 1 mmol). Yield: 224 mg (88%) of brown solid. FT-IR (ATR): ν_max_ 2806, 2589, 2126, 1633 (C=O), 1530, 1486 cm^−1^. λ_max_ (MeOH)/nm 474 (ε/dm^3^·mol^−1^·cm^−1^ 7817), 392 (9554), and 293 (19543).

#### 2.3.6. Poly{(*E*)-[4-({[4-(λ^2^-azaneyl)phenyl]amino}methylene)-6-(λ^3^-methylene)cyclohexane-1,2-dione]} (**4af**)

Prepared from **2a** (222 mg, 1 mmol) and 1,4-phenylenediamine dihydrochloride **3f** (181 mg, 1 mmol). Yield: 253 mg (99%) of purple solid. FT-IR (ATR): ν_max_ 2837, 2144, 1998, 1623 (C=O), 1515, 1442 cm^−1^. λ_max_ (MeOH)/nm 531 (ε/dm^3^·mol^−1^·cm^−1^ 873) and 293 (1530).

#### 2.3.7. Poly{(*E*)-[3-({[2-(λ^2^-azaneyl)ethyl]amino}methylene)-5-(λ^3^-methylene)cyclohexane-1,4-dione]} (**4ba**)

Prepared from **2b** (222 mg, 1 mmol) and 1,2-ethylenediamine dihydrochloride **3a** (133 mg, 1 mmol). Yield: 118 mg (56%) of black solid. FT-IR (ATR): ν_max_ 2901, 2794, 1999, 1711 (C=O), 1599, 1499, 1436, 1218, 1083, 1029, 1004, 816 cm^−1^. λ_max_ (MeOH)/nm 297 (ε/dm^3^·mol^−1^·cm^−1^ 1786) and 393 (1503).

#### 2.3.8. Poly{(*E*)-[3-({[3-(λ^2^-azaneyl)propyl]amino}methylene)-5-(λ^3^-methylene)-cyclohexane-1,4-dione]} (**4bb**)

Prepared from **2b** (222 mg, 1 mmol) and 1,2-propylenediamine dihydrochloride **3b** (147 mg, 1 mmol). Yield: 115 mg (51%) of black solid. FT-IR (ATR): ν_max_ 2977, 2886, 2686, 2042, 2026, 1712 (C=O), 1594, 1471, 1454, 1405, 1336, 1217, 1186, 1099, 1036, 957, 933, 760 cm^−1^. λ_max_ (MeOH)/nm 337 (ε/dm^3^·mol^−1^·cm^−1^ 3730) and 297 (3775).

#### 2.3.9. Poly{(*E*)-[3-({[4-(λ^2^-azaneyl)butyl]amino}methylene)-5-(λ^3^-methylene)cyclohexane-1,4-dione]} (**4bc**)

Prepared from **2b** (222 mg, 1 mmol) and 1,2-butylenediamine dihydrochloride **3c** (161 mg, 1 mmol). Yield: 89 mg (37%) of black solid. FT-IR (ATR): ν_max_ 3331, 2221, 2162, 1982, 1971, 1714 (C=O), 1554, 1436, 1222, 1023 cm^−1^. λ_max_ (MeOH)/nm 340 (ε/dm^3^·mol^−1^·cm^−1^ 1607), 297 (1573), and 251 (1764).

#### 2.3.10. Poly{(*E*)-[3-({[2-(λ^2^-azaneyl)phenyl]amino}methylene)-5-(λ^3^-methylene)cyclohexane-1,4-dione]} (**4bd**)

Prepared from **2b** (222 mg, 1 mmol) and 1,2-phenylenediamine dihydrochloride **3d** (181 mg, 1 mmol). Yield: 91 mg (35%) of black solid. FT-IR (ATR): ν_max_ 2914, 2156, 1963, 1716 (C=O), 1604, 1494, 1454, 1263, 1159, 1017, 937, 815, 749 cm^−1^. λ_max_ (MeOH)/nm 295 (ε/dm^3^·mol^−1^·cm^−1^ 3143) and 251 (1764).

#### 2.3.11. Poly{(*E*)-[3-({[3-(λ^2^-azaneyl)phenyl]amino}methylene)-5-(λ^3^-methylene)cyclohexane-1,4-dione]} (**4be**)

Prepared from **2b** (222 mg, 1 mmol) and 1,3-phenylenediamine dihydrochloride **3e** (181 mg, 1 mmol). Yield: 136 mg (53%) of black solid. FT-IR (ATR): ν_max_ 2822, 2248, 2189, 2129, 1983, 1712 (C=O), 1602, 1493, 1434, 1202, 996, 773 cm^−1^. λ_max_ (MeOH)/nm 286 (ε/dm^3^·mol^−1^·cm^−1^ 14327), 360 (7236), and 468 (5036).

#### 2.3.12. Poly{(*E*)-[3-({[4-(λ^2^-azaneyl)phenyl]amino}methylene)-5-(λ^3^-methylene)cyclohexane-1,4-dione]} (**4bf**)

Prepared from **2b** (222 mg, 1 mmol) and 1,4-phenylenediamine dihydrochloride **3f** (181 mg, 1 mmol). Yield: 209 mg (81%) of black solid. FT-IR (ATR): ν_max_ 2843, 2577, 2351, 2007, 1715 (C=O), 1509, 1367, 1174, 1058, 1015, 824 cm^−1^. λ_max_ (MeOH)/nm 346 (ε/dm^3^·mol^−1^·cm^−1^ 26115) and 292 (39256).

## 3. Results

### 3.1. Synthesis

**Scheme 1:**
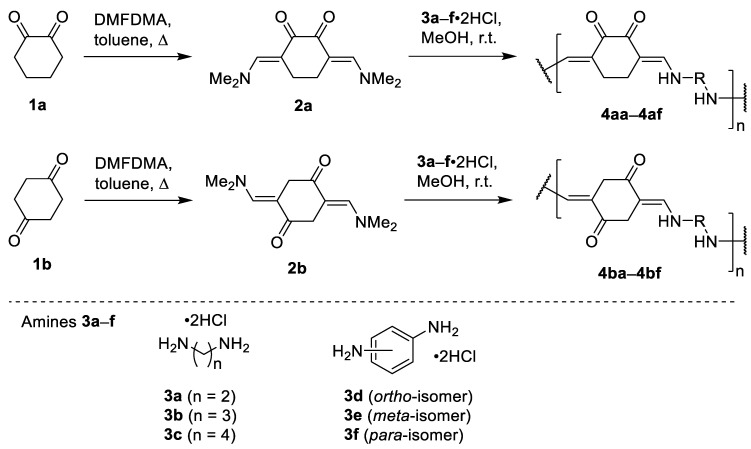
Transamination of enaminones **2a**,**b** with diamines **3a**–**f**. Synthesis of cyclohexane-1,2-dione-derived polymers **4aa**–**af** and cyclohexane-1,4-dione-derived polymers **4ba**–**bf**.

The starting bis-enaminones **2a** and **2b** were prepared in moderate yields of about 50% by heating the corresponding diketones **1a** and **1b** with 1.2 equiv. DMFDMA in toluene for 5 h. The moderate yields of enaminones **2** were due to partial conversion that could not be improved by longer reaction time and higher reaction temperature (Scheme 1). Bis-enaminones **2a** and **2b** were then treated with equimolar amounts of aliphatic (**3a**–**c**) and aromatic diamines (**3d**–**f**) dihydrochlorides in methanol at room temperature for 72 h and the precipitated products **4** were obtained by filtration in 10–99% yields (Scheme 1, Table 1).

**Scheme 2:**
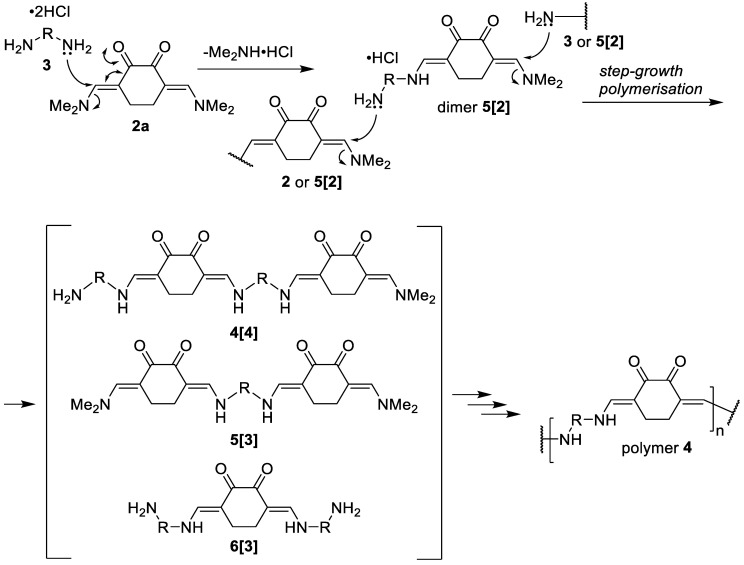
The proposed pathway of step-wise polymerisation through oligomeric intermediates **4**[**n**], **5**[**n**], **6**[**n**].

According to previously reported closely related transformations, reactions of bis-enaminones **2** and diamines **3** most probably proceed as a step-growth polymerisation, where consecutive intermolecular transaminations give polymeric products **4** [19,20,29]. The proposed reaction pathway is shown in Scheme 2. Acid-catalyzed transamination of bis-enaminone **2** with one equivalent of diamine **3** gives a bifunctional dimer **5[2]**, while further reaction of **5[2]** can take place in three ways. Reaction of **5[2]** with another dimer **5[2]** gives oligomeric intermediate **4[4]**, reaction of **5[2]** with enaminone **2** gives the oligomer **5[3]**, while reaction of **5[2]** with diamine **3** gives the oligomer **6[3]**. Subsequent transaminations of the intermediates **4**–**6** with monomers **2** and **3** or between the oligomeric intermediates **4**–**6** results in polymer **4**.

### 3.2. Characterisation

The monomers **2a** and **2b** were characterised by spectroscopic methods (^1^H NMR, ^13^C NMR, FTIR, and HRMS) and by elemental analysis for C, H, and N. The products **4aa**–**4af**, and **4ba**–**4bf** were characterised by spectroscopic methods (^1^H NMR, FTIR, and UV-vis), size-exclusion chromatography (SEC), thermogravimetric analysis-mass spectromety (TGA-MS), and scanning electron microscopy (SEM).

FTIR spectra of compounds **4** show absorption bands at around 1600 cm^−1^, which are characteristic for the C=O group of a conjugated ketone. Absorption bands around 2900 cm^−1^ in the spectra of the enaminones **2a** and **2b**, were in line with typical C–H and N–H absorption bands [30,31,32,33,34]. Broad absorption bands around 3000 cm^−1^ in the spectra of compounds **4** were in agreement with N–H···O=C hydrogen-bonding of compounds **4** in the solid state. For details see the Supplementary Material.

**Figure 2:**
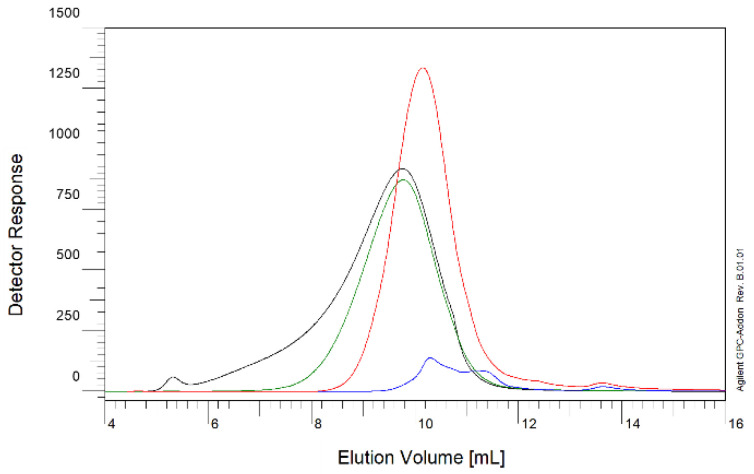
SEC-UV chromatograms of compounds **4aa** (red line), **4af** (blue line), **4ba** (green line), and **4bc** (black line). Sample **4af** was only partially soluble in TEAN/HFIP.

Molar mass characteristics were determined by relative SEC in TEAN/HFIP for the representative samples **4aa**, **4af**, **4ba**, and **4bc** (Figure 2, Table 2). Sample **4aa** (▬) shows molar mass averages typical for oligomeric species with the respective dispersity of 1.44 (Table 2; entry 1). Sample **4af** (▬) was almost insoluble in TEAN/HFIP, therefore the results for this sample are not representative (Figure 2; Table 2; entry 2). On the other hand, compounds **4ba** (▬) and **4bc** (▬) show much higher molar mass averages typical for polymeric species with dipersity values of 1.91 and 5.29, respectively (Figure 2, Table 2, entries 3 and 4). These results support a step-growth polymerization mechanism showing 1,4-disubstituted bis-enaminone **1b** (Table 2; entries 3 and 4) undergoes significantly higher degree of polymerisation than the 1,2-disubstituted bis-enaminone analogue **1a** (Table 2; entry 1).

UV-vis spectra of compounds **4** were measured in MeOH at 250–800 nm and showed absorption maxima at around 300, 350, and 450 nm (for details see the Supplementary Material), which was consistent with the data for closely related polymers [19,20]. These UV-vis spectral data of compounds **4** indicated interesting UV light shielding properties that might find use in various optical applications. 

**Figure 3:**
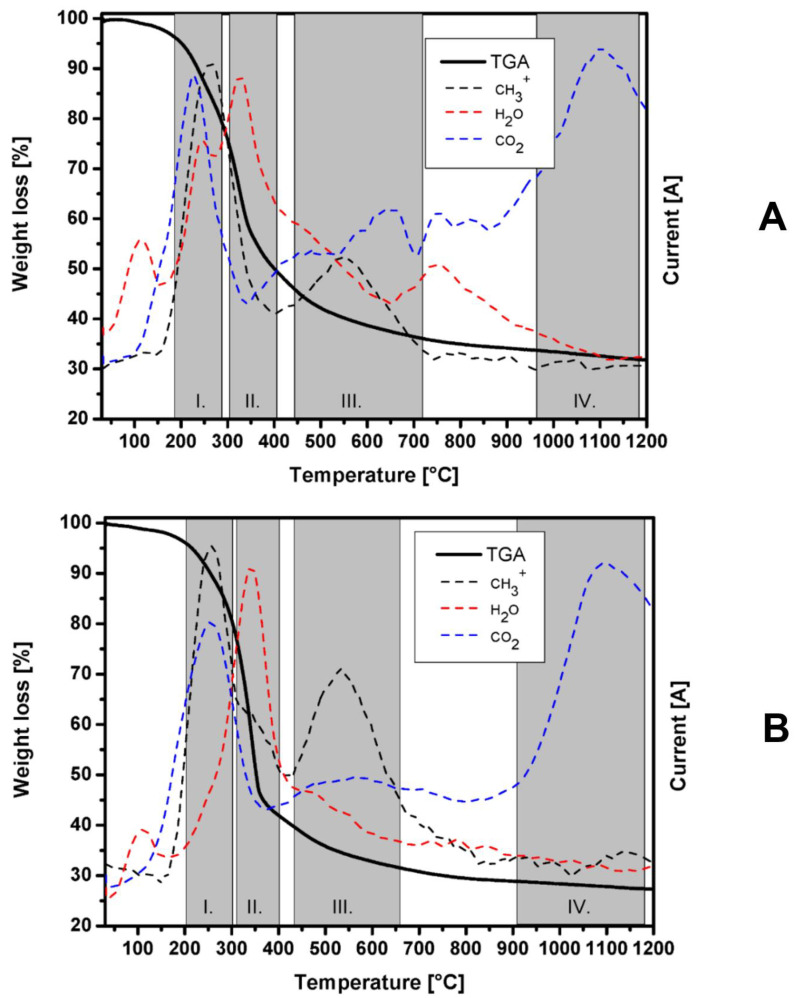
Evolved gas analysis of polyenaminone **4aa** (**A**) and polyenaminone **4ba** (**B**). Different colors represent fragments with *m*/*z* that correspond to methyl (CH_3_^+^), water and CO_2_ signals. Black curves represent the TGA profiles of polyenaminones **4aa** and **4ba**. Gray rectangles represent region I, region II, region III, and region IV respectively.

**Figure 4:**
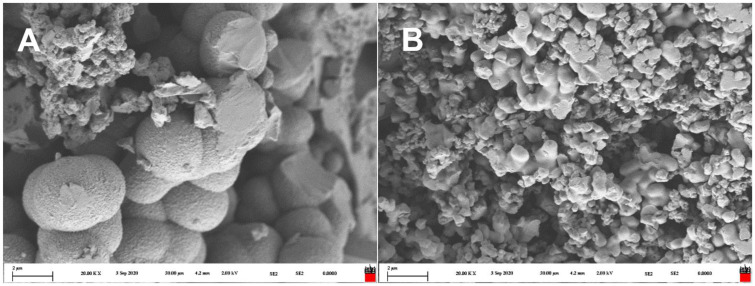
SEM images showing morphology of polymers **4aa** (**A**) and **4ba** (**B**) taken at 2 × 10^4^ magnification.

Compounds **4** did not exhibit melting points between 20 and 300 °C. They were thermally stable up to 200 °C and then underwent decomposition above 200 °C. This was not really surprising, as similar absence of melting points is also characteristic for related polyenaminones [19,20]. The representative polymers **4aa** (Figure 3A) and **4ba** (Figure 3B) were characterised for thermal properties using thermogravimetric analysis [35]. Polyenaminones **4aa** and **4ba** were pyrolysed in Ar atmosphere in order to probe their thermal stability in inert environments relevant to battery applications. Thermogravimetric curves (Figure 3A,B) revealed that both of polyenaminones behave similarly under elevated temperatures. They start to decompose at temperature above 200 °C, which exhibit high stability of polyenaminones for potential battery applications. The acceptable operating temperature region for Li-ion batteries is between −20 °C and +60 °C. Total weight loss over the entire temperature range is 68.2 wt.% and 72.3 wt.% for **4aa** and **4ba**, respectively. Further, closer inspection of both thermograms (Figure 3A,B) revealed that the decomposition of polyenaminones happens in at least 4 different temperature regions. Regions were defined arbitrary according to *m*/*z* fragments that represent methyl (CH_3_^+^), water and CO_2_ signals. In the first region (temperature range between 200 °C and 300 °C), evolution of all three species are released as decomposition products. In the second region (temperature range between 300 °C and 400 °C), where the highest weight loss occurs, CH_3_^+^ and water are released. Third region (temperature range between 450 °C and 700 °C) represents evolution of CH_3_^+^ and fourth (temperature range between 950 °C and 1200 °C) only CO_2_ is evolving. Regions I and II are related to thermal degradation (pyrolysis) of polyenaminones, while regions III and IV are related to decomposition of resilient functional groups and graphitization of the carbon residue. We also speculate that pyrolyzed polyenaminones can have potential use in electrocatalysis since they are expected to have nitrogen doped in graphitized structure.

Polymers **4aa** and **4ba** were morphologically analysed using SEM (Figure 4) [1,2,3,4,36,37]. For both polymers, SEM images were taken at the same magnification (2 × 10^4^) and compared. The morphological analysis revealed that both polymers are agglomerates consisting of smaller particles with irregular shapes and a broad particle size distribution. Polymer **4aa** (Figure 4A) consists of agglomerated particles with a diameter up to 3 μm, while the particles of polymer **4ba** (Figure 4B) are smaller and have a diameter up to 0.5 μm. Moreover, the morphology of the particles of both polymers exhibits a porous structure, which is of great importance if these materials are to be used in energy storage devices such as batteries. High porosity and the associated high specific surface area are important for the accessibility of the battery electrolyte and thus for achieving high current densities. This could have a major impact on the lifetime and operation of the batteries.

**Figure 5:**
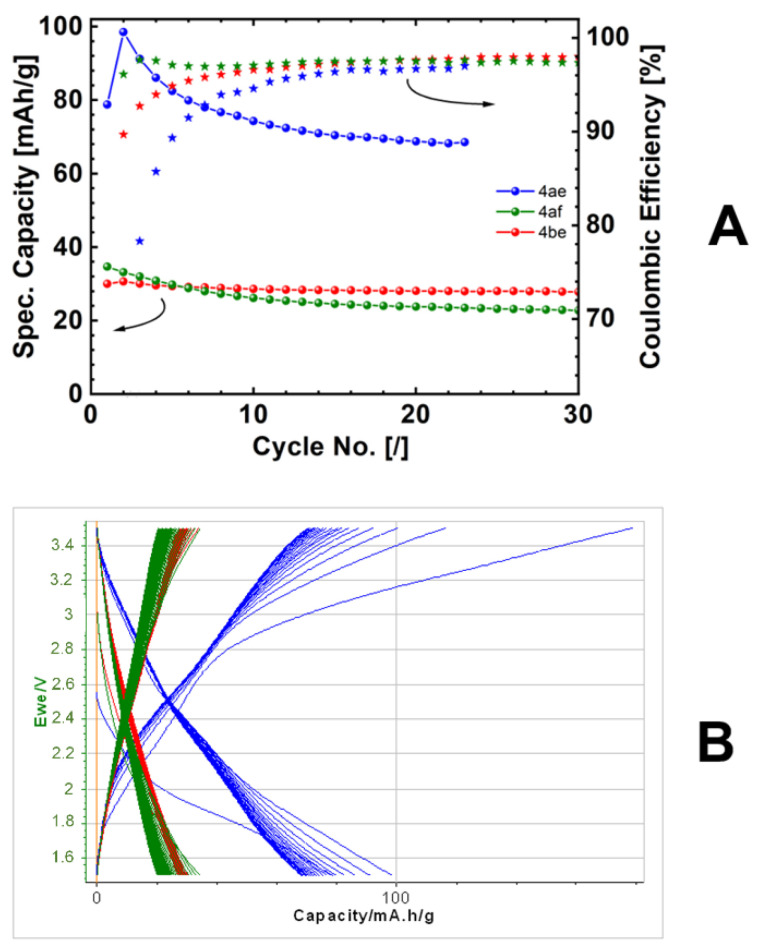
Capacity (full line) and Coulombic efficiency (stars) of compounds **4ae** (▬), **4af** (▬), and **4be** (▬) vs. cycle number (**A**). Galvanostatic curves of compounds **4ae** (▬), **4af** (▬), and **4be** (▬) (**B**).

Redox activity of compounds **4ae**, **4af**, and **4be** was investigated in Li-battery, where polymers were used as a cathode materials and Li metal as anode. From galvanostatic tests capacity vs. cycle number was extracted, as shown in Figure 5A. The compound **4ae** exhibited maximal 98 mAh/g capacity which quickly dropped but then stabilized at 68 mAh/g after 20 cycles. This capacity drop can be attributed to limited solubility of **4ae** polymer inside ethereal electrolyte and/or side reactions in initial cycles, which are both known phenomena from the literature [38]. Material **4ae** showed low Coulombic efficiency <95% in first 10 cycles, which could further indicate side electrochemical reactions, but then it stabilized at around 97%. On the other hand, compounds **4af** and **4be** showed low capacity, which is probably connected only to carbon black pseudocapacity and not to real redox activity of the polymers (Figure 5A). The galvanostatic curves of compounds **4ae**, **4af**, and **4be** are presented in Figure 5B. The curves did not exhibit any discernible plateaus. Nevertheless, the slope of **4ae** (▬) changes, which can indicate redox activity.

**Figure 6:**
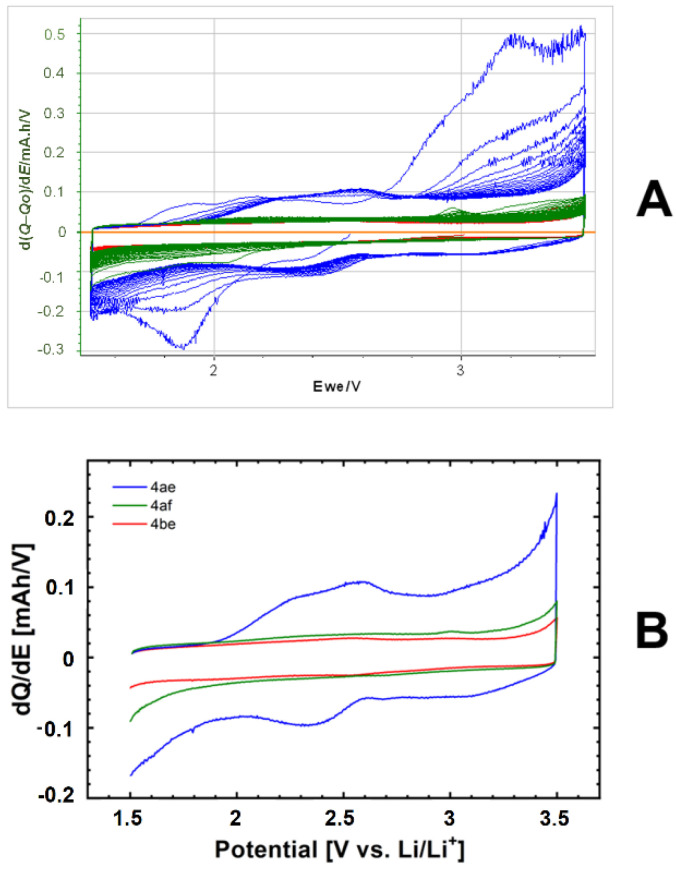
d*Q*/d*E* curves of compounds **4ae** (▬), **4af** (▬), and **4be** (▬). (**A**) and d*Q*/d*E* curves of compounds **4ae** (▬), **4af** (▬), and **4be** (▬) after the 10th cycle (**B**).

To study electroactivity more in detail, d*Q*/d*E* vs. potential E is presented in Figure 6A. In the curve of compound **4ae** (▬), peaks 3.20 V (Oxidation) and 1.8 V vs. Li/Li^+^(Reduction) diminish after few cycles and are probably linked to irreversible reactions (low Coulombic efficiency). The other two compounds exhibited almost flat curves (▬, ▬), indicating pseudocapacity without redox activity (Figure 6A). The origin of pseudocapacity is high surface area of electron-conductive carbon black additive, which is needed for electrode preparation. After the 10th cycle, d*Q*/d*E* curve of compound **4ae** (▬) exhibited two oxidation peaks at 2.32 V and 2.58 V vs. Li and one reduction peak at 2.32 V vs. Li (Figure 6B). These potentials are in agreement with other carbonyl materials [39], which generally show voltages of 2.0–3.0 V vs. Li/Li^+^.

**Figure 7:**
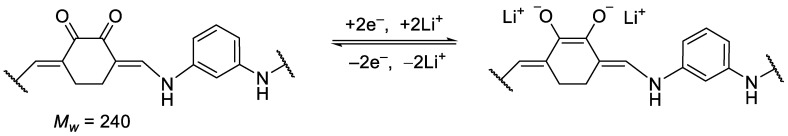
Proposed redox reaction of **4ae** material during charge/discharge inside Li-battery.

Although compound **4ae** doesn’t contain real *ortho*-quinone group, it can be still regarded as 1,2-diketone and can act as a redox centre [40]. Proposed redox reaction is presented in Figure 7. Theoretical capacity of **4ae** is 223 mAh/g, according to proposed two electron redox reaction and Equation (1). The maximum obtainable capacity was 98 mAh/g, which is around 44% of the theoretical value. Such low values of material utilization are common [39,41,42] in Li-organic batteries and are usually due to limited ionic and electronic transport inside organic materials (electrical isolators). Capacity could be further improved by preparation of smaller particles [43,44], composites with electron-conductive additives [45,46], electrode and electrolyte engineering [47] but is not main topic of this article. Another interesting observation is that compound **4af**, which is very similar to **4ae** with the same 1,2-diketone redox centre, doesn’t show any redox activity. The reason could be in a much worse ionic and electronic transport of **4af** material: bigger particles, lower porosity, more rigid structure of the polymer, etc. It is also possible that redox activity of **4be** with different 1,4-diketone centre is hindered due to the same reason.

(1) $$C_{theo}=\frac{z\cdot F}{M_{W}}$$

where *C_theo_*…theoretical capacity [mAh/g]; *z*…number of exchanged electrons [/]; *F*…Faraday constant [96485 As/mol = 26801 mAh/mol]; *M_W_*…Molecular weight of monomer unit [240 g/mol].

## 4. Conclusions

Acid-catalysed transamination of bis-enamino ketones **2a** and **2b** with aliphatic (**3a**–**c**) and aromatic (**3d**–**f**) diamines gave polyenaminones **4aa**–**4af** and **4ba**– **4bf** in 10–100% yields with molar mass averages from around 7 to 50 kg/mol. Polyenaminones **4aa**–**4af** and **4ba**–**4bf** are practically insoluble in conventional organic solvents. Monomer building blocks **2a**,**b** and **3a**–**f** are accessible from biomass-derived precursors, such as cyclohexanone, succinic acid, and alkanediols. Compounds of group **4** strongly absorb UV light at wavelengths below 500 nm, indicating their promising potential in optical and optoelectronic applications. Preliminary experiments showed that some of these polymers are redox active molecules, which could be used in energy storage devices. However, further investigation needs to be done with polyenaminones bearing quinone and hydroquinone redox centers to fully exploit their theoretical capacities.

## Figures and Tables

**Table 1:** Experimental data for compounds **4**.

Entry	Transformation	Yield (%) ^1^	Entry	Transformation	Yield (%) ^1^
1	**2a + 3a → 4aa**	87	7	**2b + 3a → 4ba**	56
2	**2a + 3b → 4ab**	10	8	**2b + 3b → 4bb**	51
3	**2a + 3c → 4ac**	13	9	**2b + 3c → 4bc**	37
4	**2a + 3d → 4ad**	48	10	**2b + 3d → 4bd**	35
5	**2a + 3e → 4ae**	88	11	**2b + 3e → 4be**	53
6	**2a + 3f → 4af**	99	12	**2b + 3f → 4bf**	81

^1^ Isolated yield.

**Table 2:** Molar mass characteristics of the representative compounds **4aa**, **4af**, **4ba**, and **4bc**.

Entry	Compound	*M_w_* (g/mol)	*M_n_* (g/mol)	*M_w_*/*M_n_*
1	**4aa**	6900	4800	1.44
2	**4af ^1^**	- ^1^	- ^1^	- ^1^
3	**4ba**	14300	7500	1.91
4	**4bc**	49700	9400	5.29

^1^ Poorly soluble sample.

## Data Availability

The data presented in this study are available in the main manuscript and in the Supplementary Materials of this manuscript.

## Supplementary Materials

The following are available online at https://www.mdpi.com/article/10.3390/polym14194120/s1, copies of ^1^H NMR, ^13^C NMR, IR, and UV-vis spectra of compounds **2a**, **2b**, **4aa**–**4af**, and **4ba**–**4bf**.
