# Supplementary material for: Synthesis of Optically and Redox Active Polyenaminones from Diamines and α,α’-Bis[(dimethylamino)methylidene]cyclohexanediones
Source: Polymers (Basel). 2022 Oct 1;14(19):4120. doi: 10.3390/polym14194120 (PMC9573701; doi:10.3390/polym14194120)
Supplement: Supplementary file 1 [file polymers-14-04120-s001.zip › polymers-1914668-supplementary.pdf]

polymers

MDPI

Supplementary material

Synthesis of Optically and Redox Active Polyenaminones from Diamines and α,α'-Bis[(dimethylamino)methylidene]cyclohexanediones.

Urša Štanfel [1], Tomaž Kotnik [1], Sebastijan Ričko [1], Uroš Grošelj [1], Bogdan Štefane [1], Klemen Pirnat [3], Ema Žagar [2], Boštjan Genorio [1]* and Jurij Svete [1,*]

[1] University of Ljubljana, Faculty of Chemistry and Chemical Technology, Večna pot 113, 1000 Ljubljana, Slovenia
[2] National Institute of Chemistry, Department of Materials Chemistry, Hajdrihova 19, 1000 Ljubljana, Slovenia
[3] National Institute of Chemistry, Department of Polymer Chemistry and Technology, Hajdrihova 19, 1000 Ljubljana, Slovenia

Table of contents

1. Copies of ^{1}H NMR and ^{13}C NMR Spectra of Compounds **2** and **4**. 2–4

2. Copies of IR spectra of compounds **2** and **4**. 5–13

3. Copies of UV-VIS spectra of compounds **4aa**–**4af** and **4ba**–**4bf**. 9–14

1. Copies of ^{1}H NMR and ^{13}C NMR Spectra of Compounds 2 and 4.

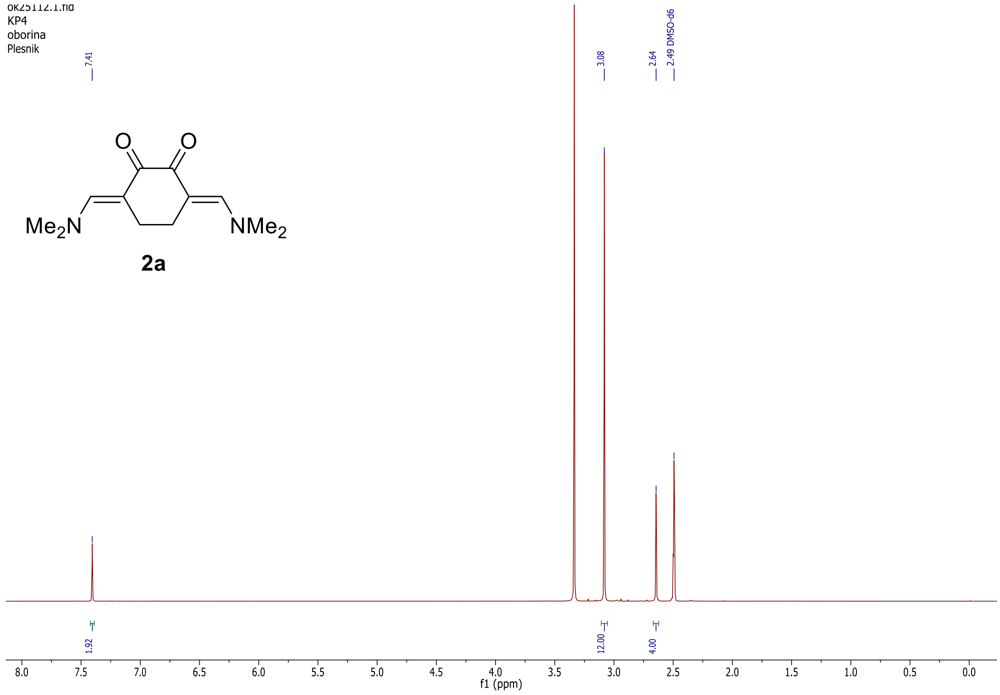

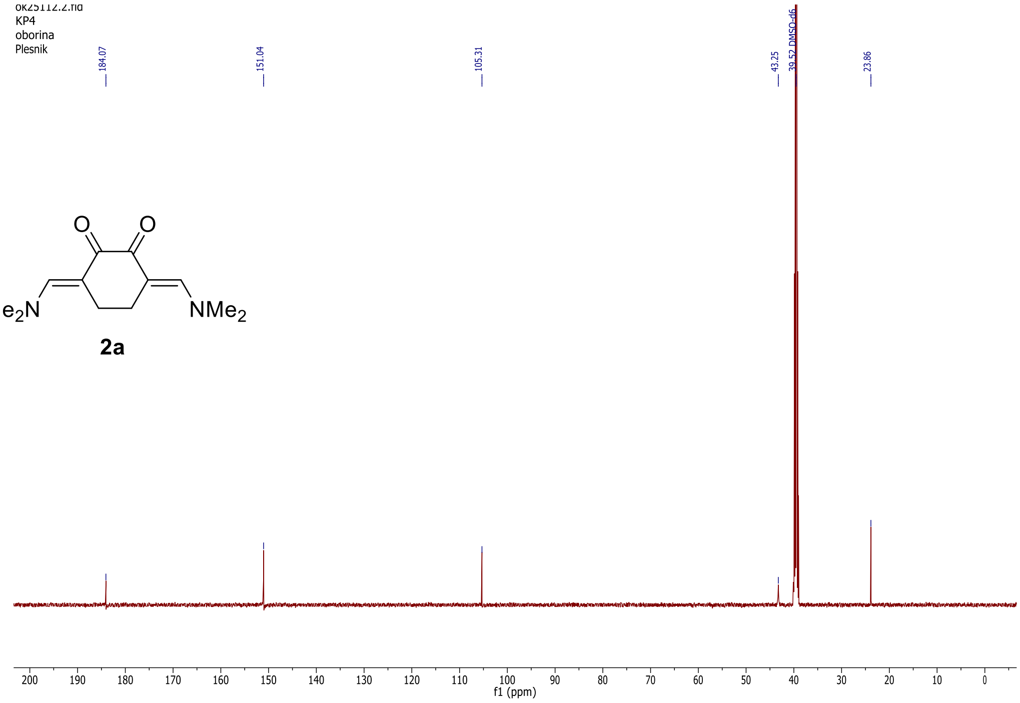

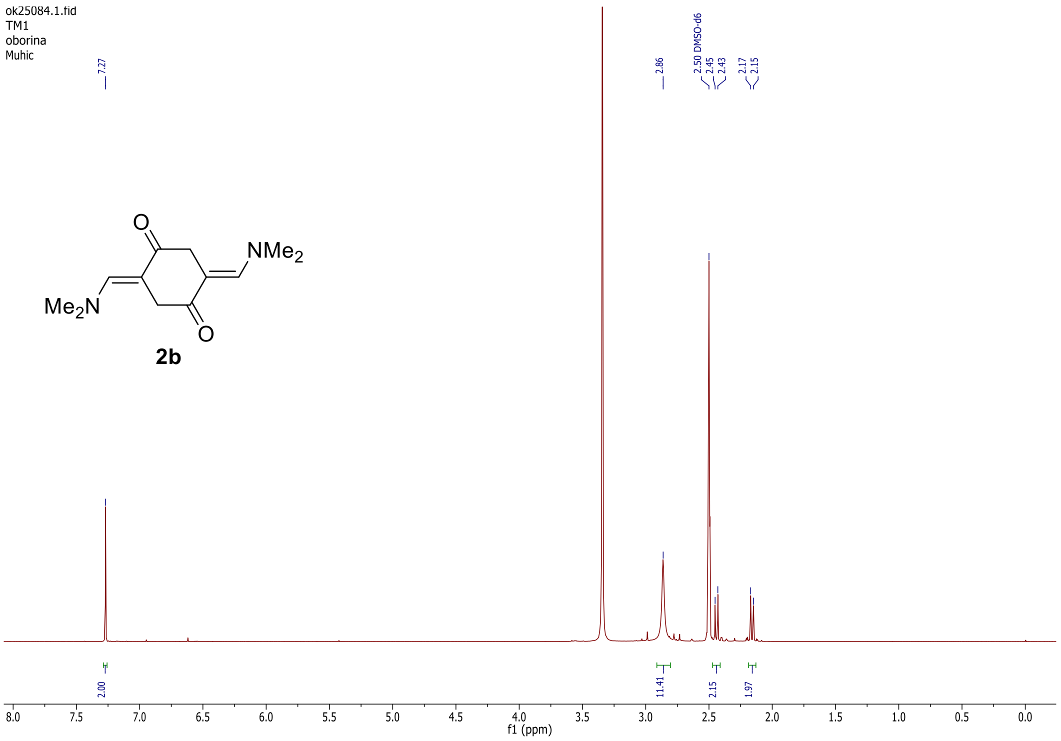
ok25084.1.fid
TM1
oborina
Muhic
7.27
2.86
2.50 DMSO-d6
2.45
2.43
2.17
2.15
NMe2
Me2N
2b
2.00
11.41
2.15
1.97
f1 (ppm)

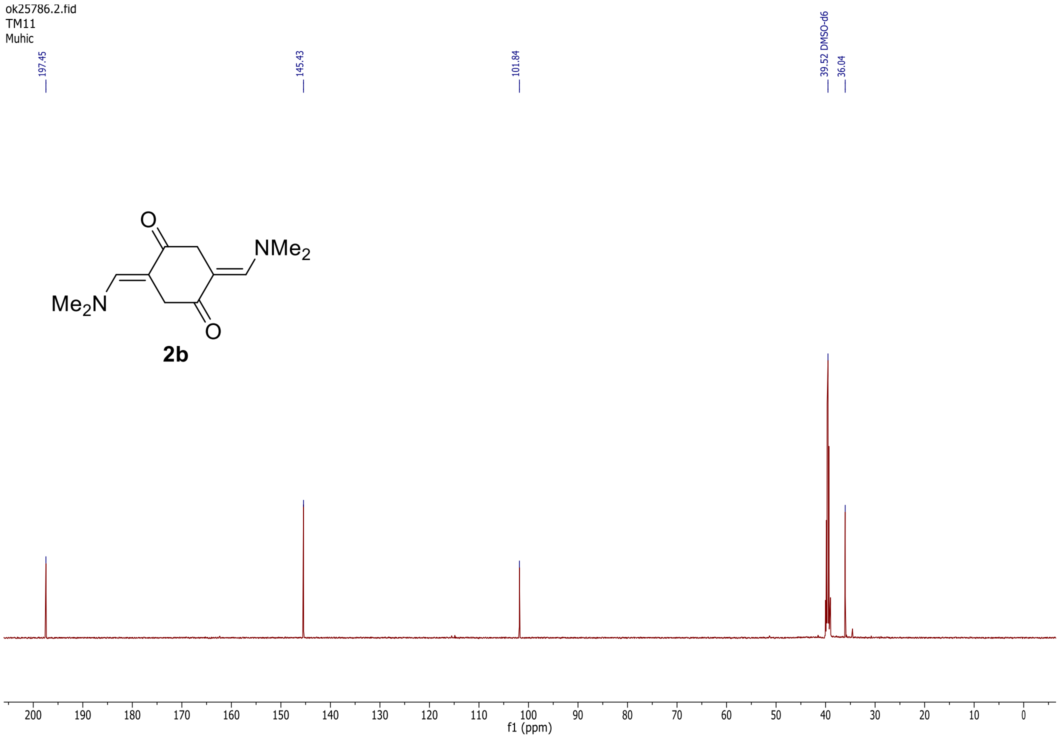
ok25786.2.fid
TM11
Muhic
197.45
145.43
101.84
39.52 DMSO-d6
36.04
NMe2
Me2N
2b
f1 (ppm)

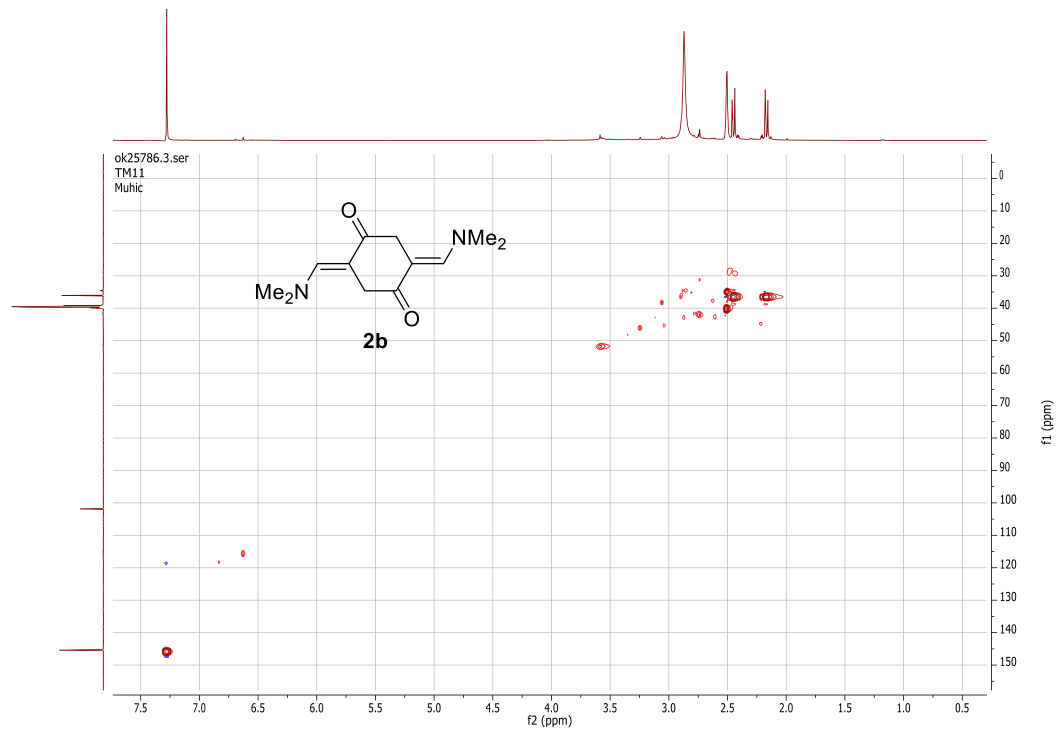
ok25786.3.ser
TM11
Muhic
O
NMe2
Me2N
O
2b
f1 (ppm)
f2 (ppm)

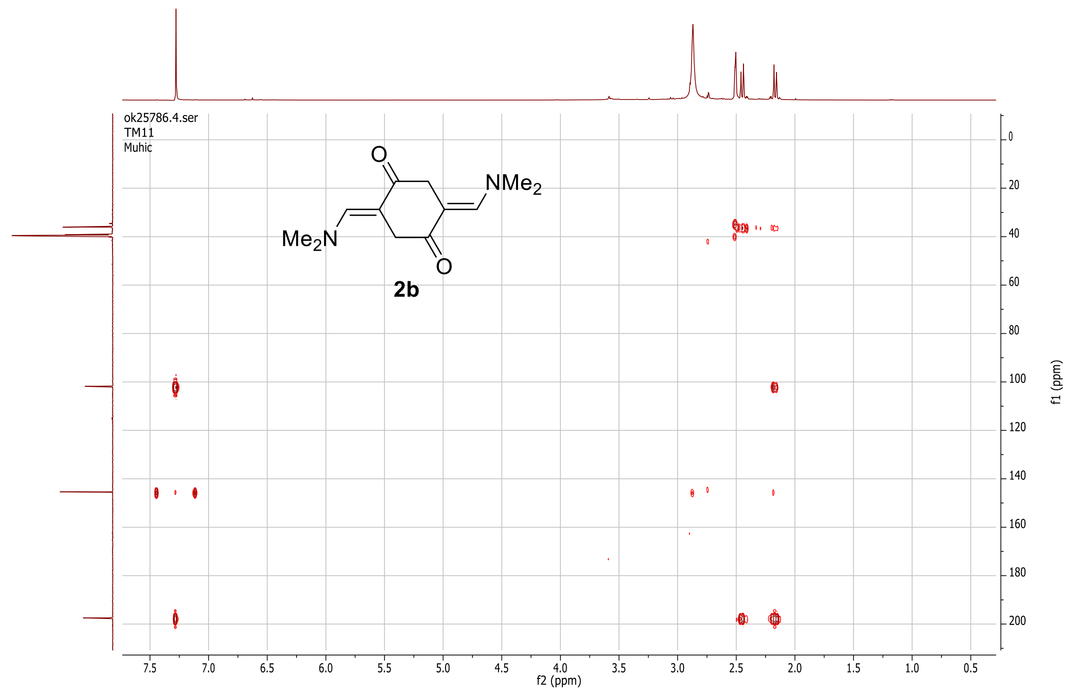
ok25786.4.ser
TM11
Muhic
O
NMe2
Me2N
O
2b
f1 (ppm)
f2 (ppm)

2. Copies of IR spectra of compounds 2 and 4.

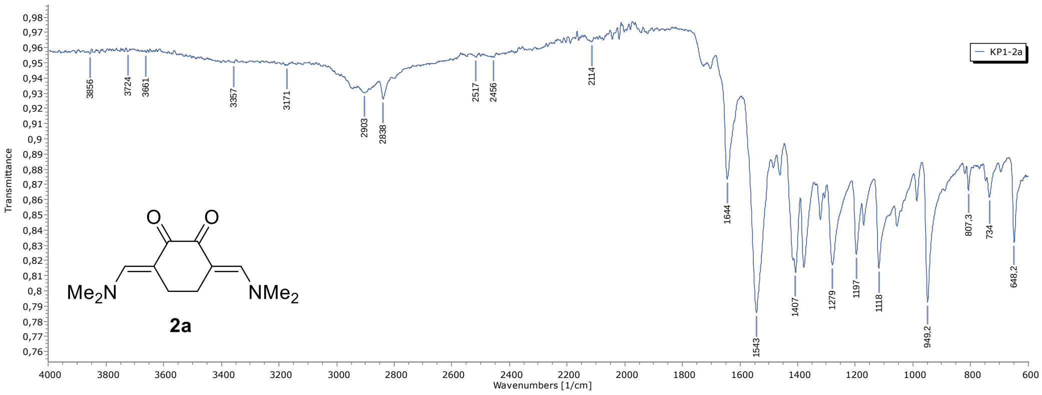

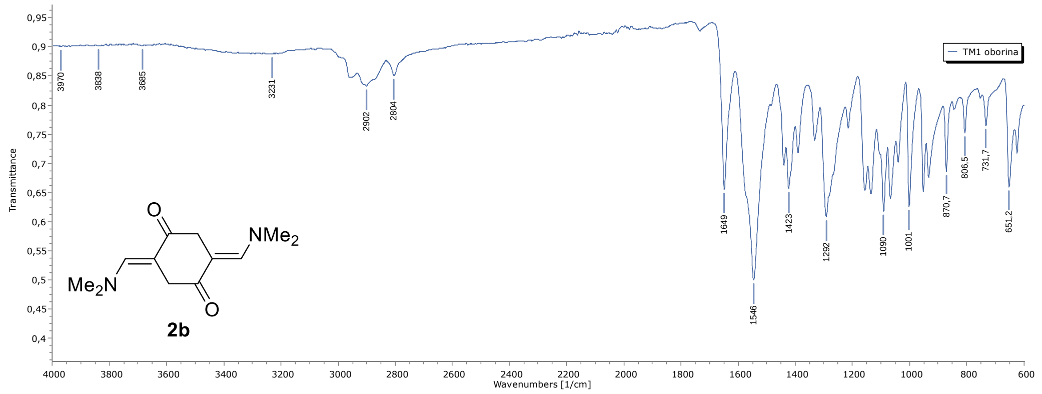

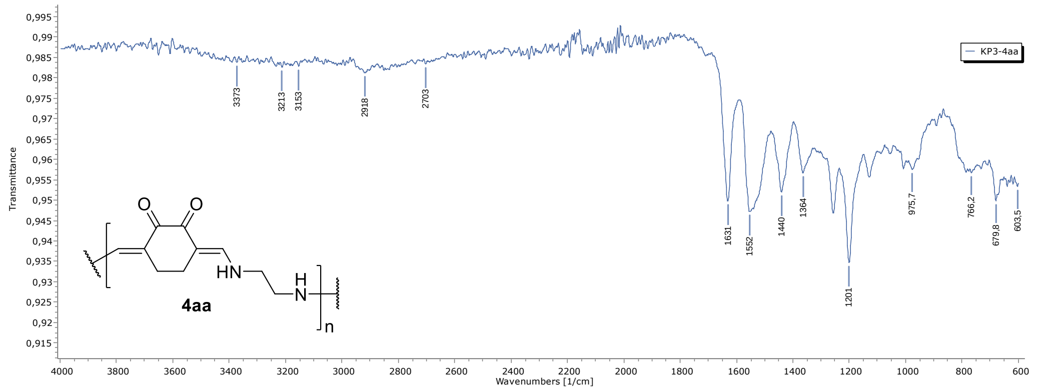

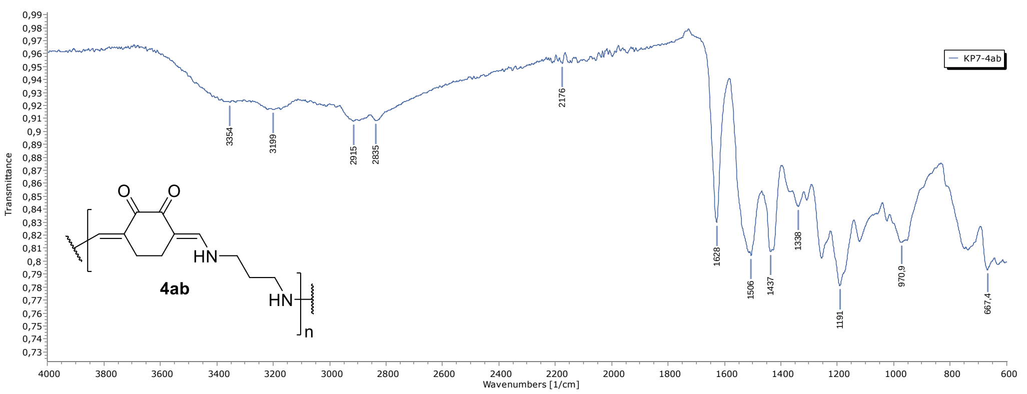

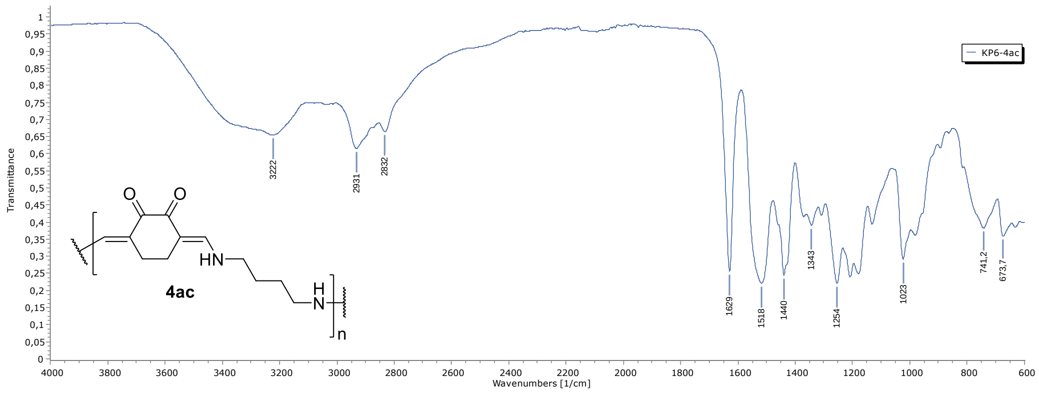
KP6-4ac
4ac
Transmittance
Wavenumbers [1/cm]
3222
2931
2832
1629
1518
1440
1343
1254
1023
741,2
673,7

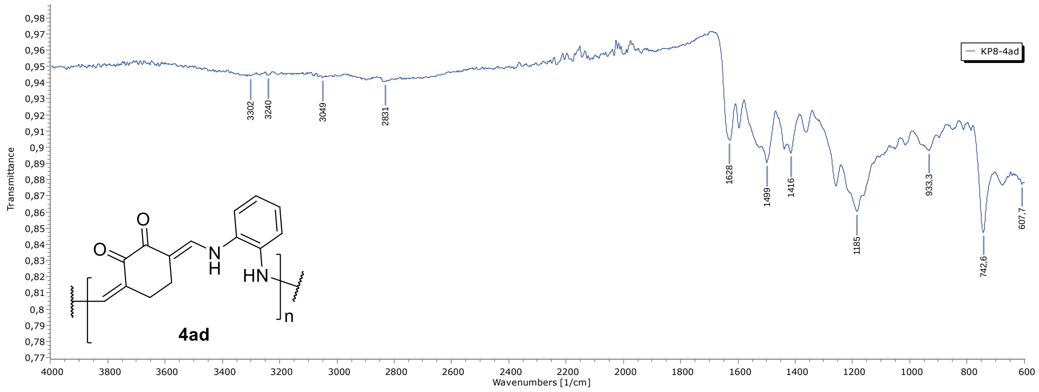
KP8-4ad
4ad
Transmittance
Wavenumbers [1/cm]
3302
3240
3049
2831
1628
1499
1416
1185
933,3
742,6
607,7

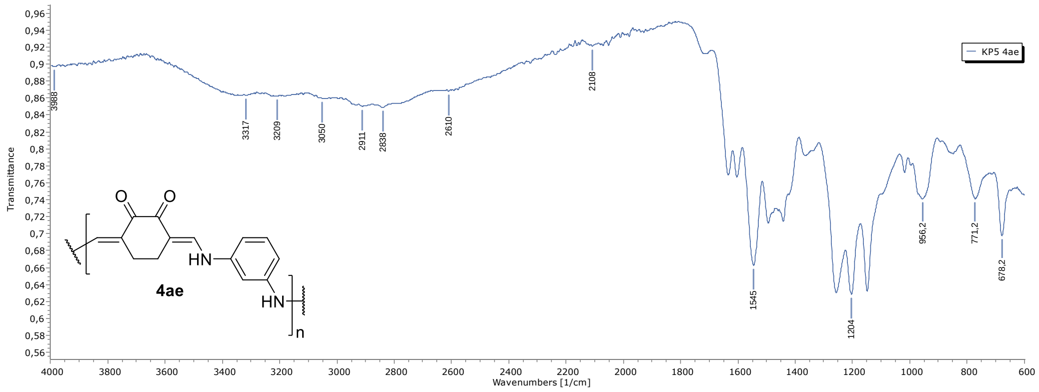
KP5 4ae
4ae
Transmittance
Wavenumbers [1/cm]
3988
3317
3209
3050
2911
2838
2610
2108
1545
1204
956,2
771,2
678,2

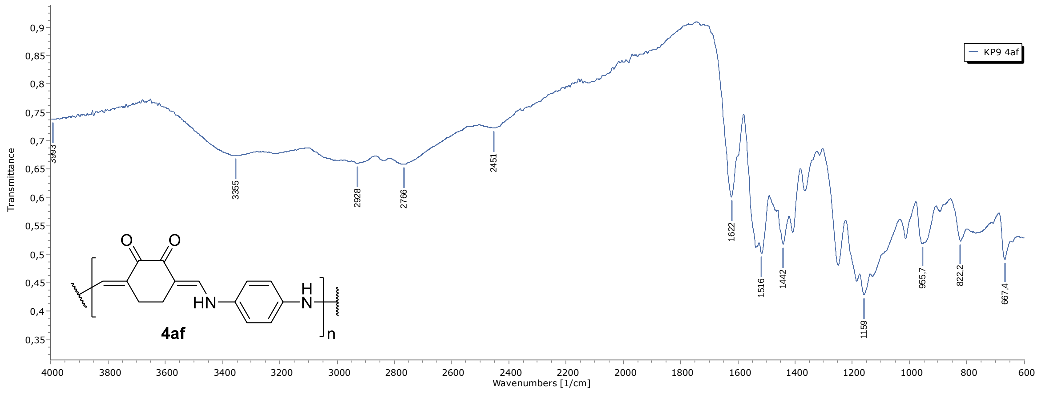
KP9 4af
4af
Transmittance
Wavenumbers [1/cm]
3993
3355
2928
2766
2451
1622
1516
1442
1159
955,7
822,2
667,4

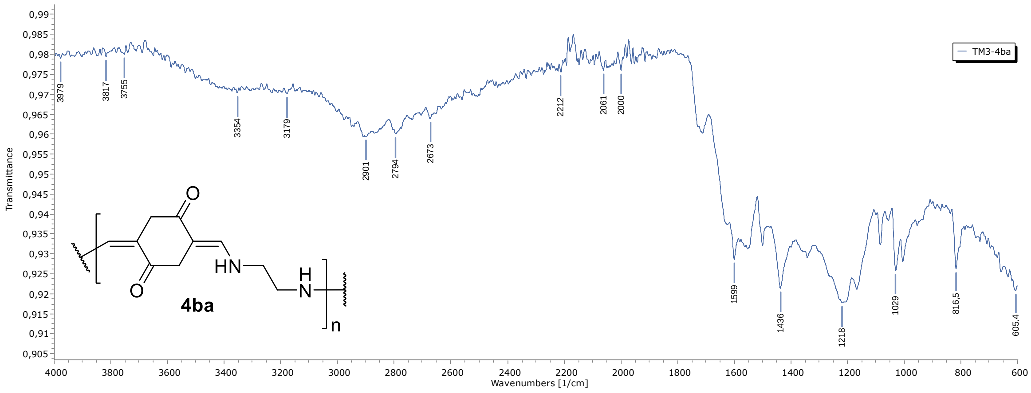
TM3-4ba
Transmittance
Wavenumbers [1/cm]
3979
3817
3755
3354
3179
2901
2794
2673
2212
2061
2000
1599
1436
1218
1029
816,5
605,4
4ba

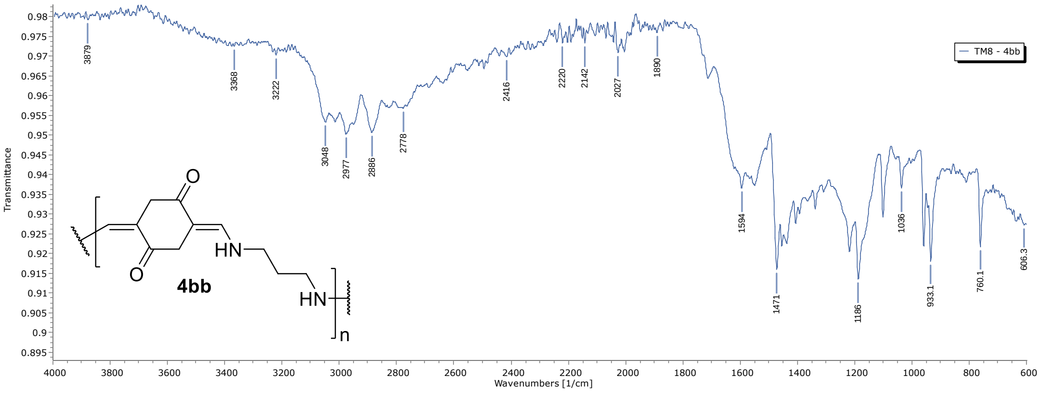
TM8 - 4bb
Transmittance
Wavenumbers [1/cm]
3879
3368
3222
3048
2977
2886
2778
2416
2220
2142
2027
1890
1594
1471
1186
1036
933.1
760.1
606.3
4bb

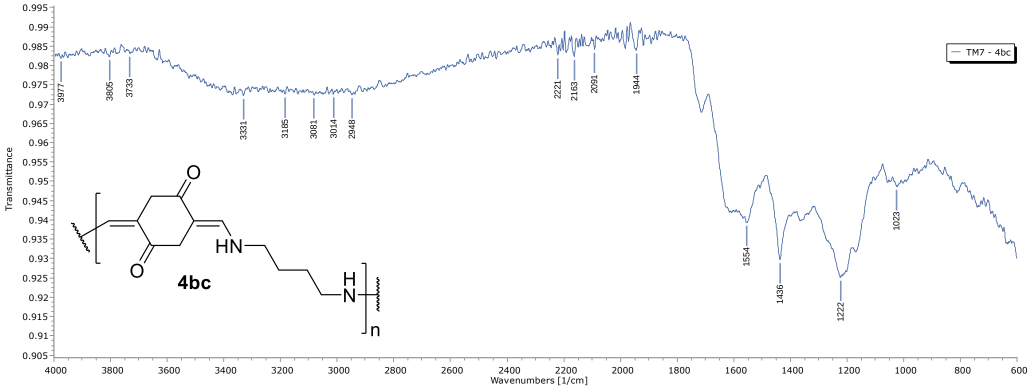
TM7 - 4bc
Transmittance
Wavenumbers [1/cm]
3977
3805
3733
3331
3185
3081
3014
2948
2221
2163
2091
1944
1554
1436
1222
1023
4bc

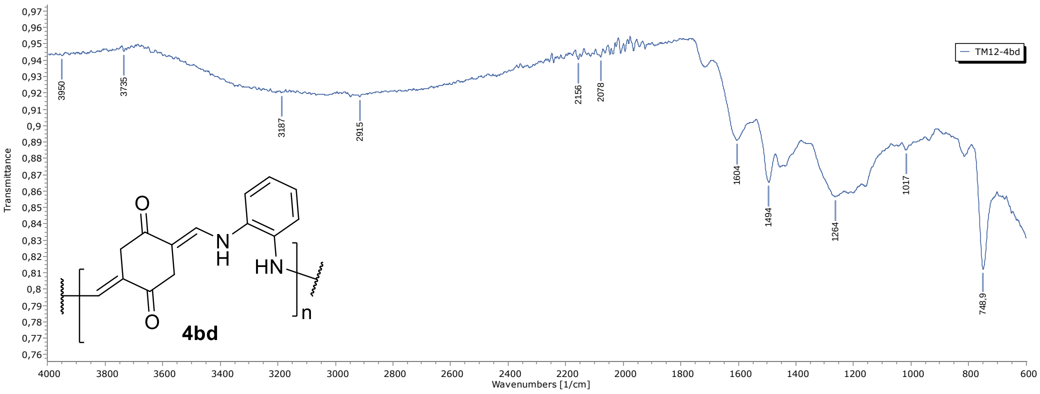
TM12-4bd
Transmittance
Wavenumbers [1/cm]
3950
3735
3187
2915
2156
2078
1604
1494
1264
1017
748,9
4bd

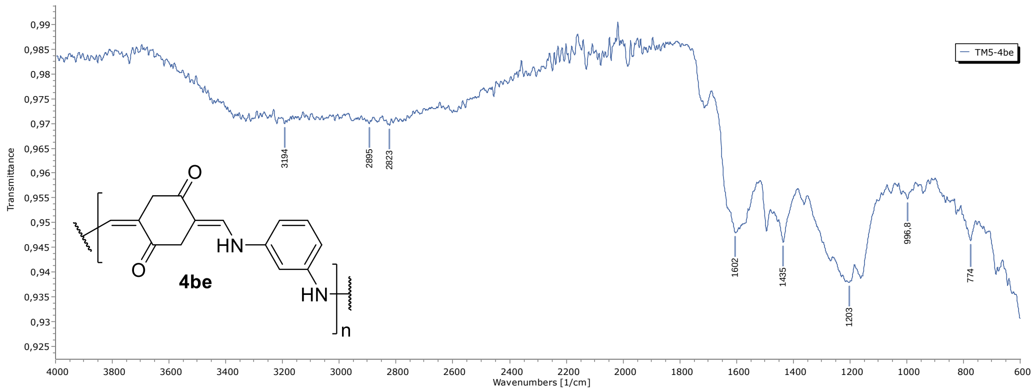
TM5-4be
Transmittance
Wavenumbers [1/cm]
3194
2895
2823
1602
1435
1203
996,8
774
4be

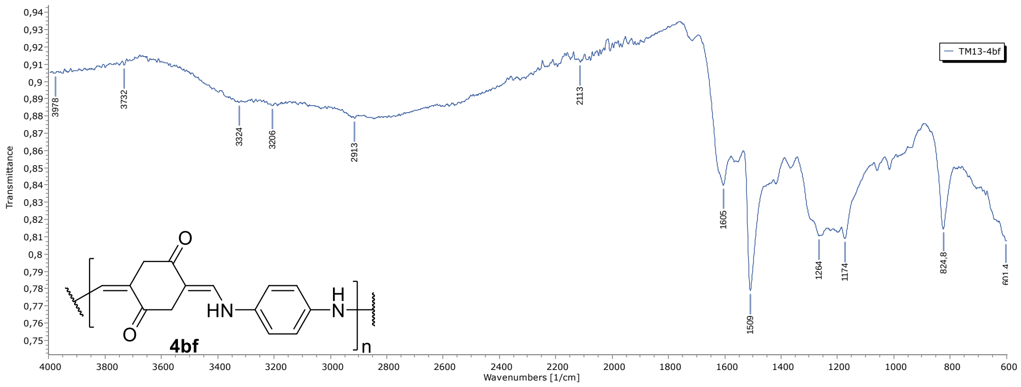
TM13-4bf
Transmittance
Wavenumbers [1/cm]
3978
3732
3324
3206
2913
2113
1605
1509
1264
1174
824,8
601,4
4bf

3. Copies of UV-VIS spectra of compounds 4aa–4af and 4ba–4bf.

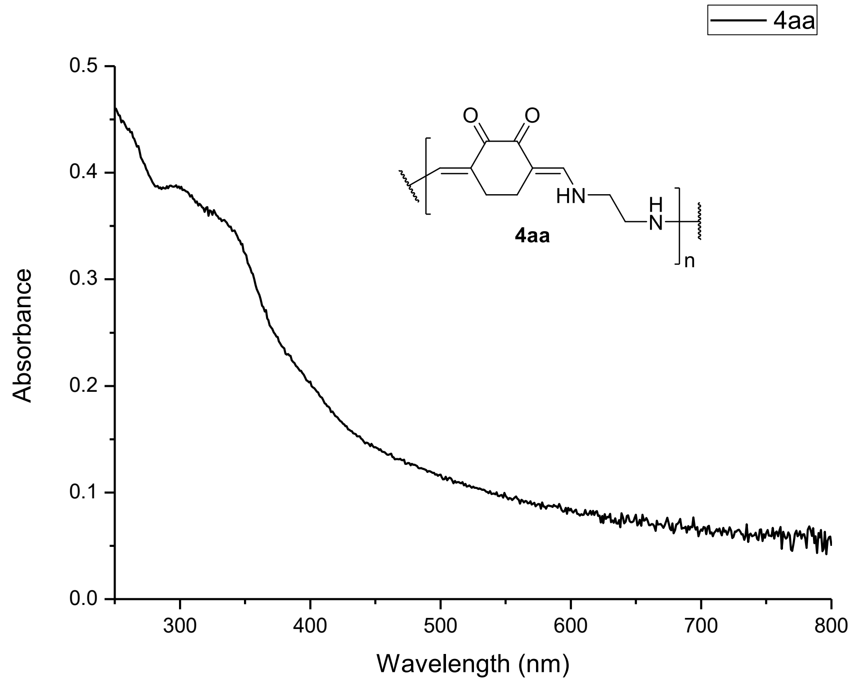

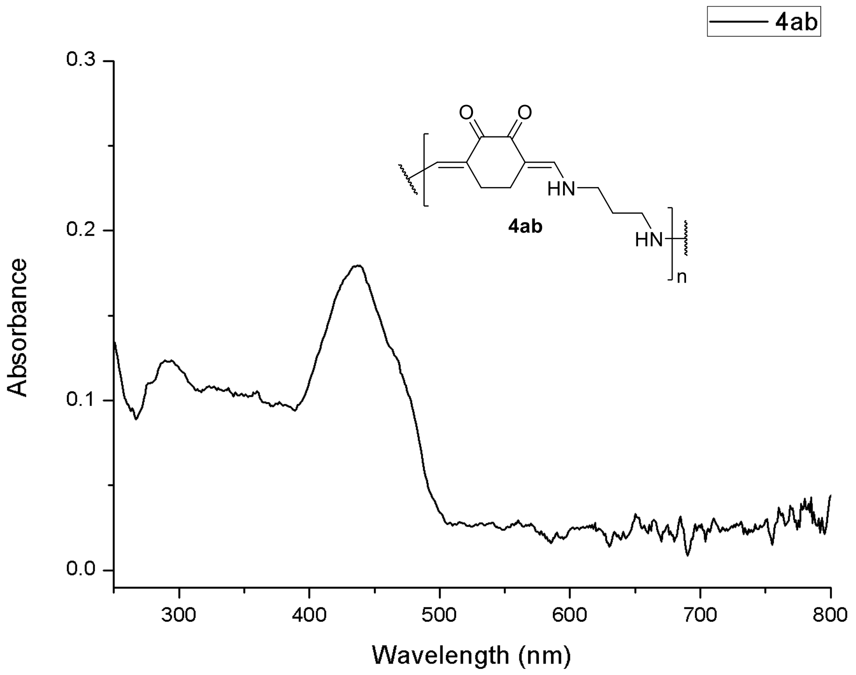

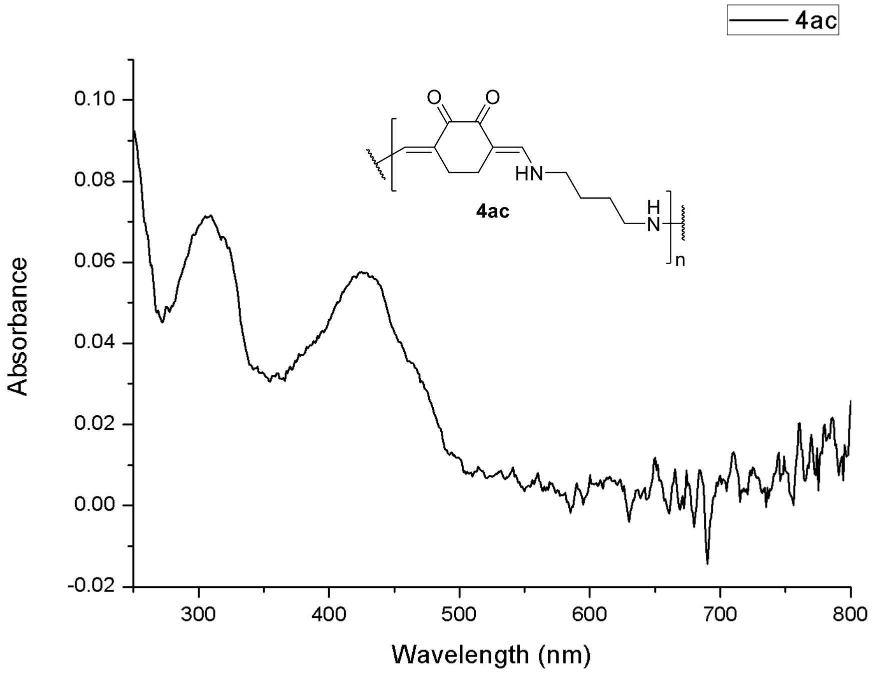
4ac
Absorbance
Wavelength (nm)
4ac
HN
H
N
n
O
O
0.10
0.08
0.06
0.04
0.02
0.00
-0.02
300
400
500
600
700
800

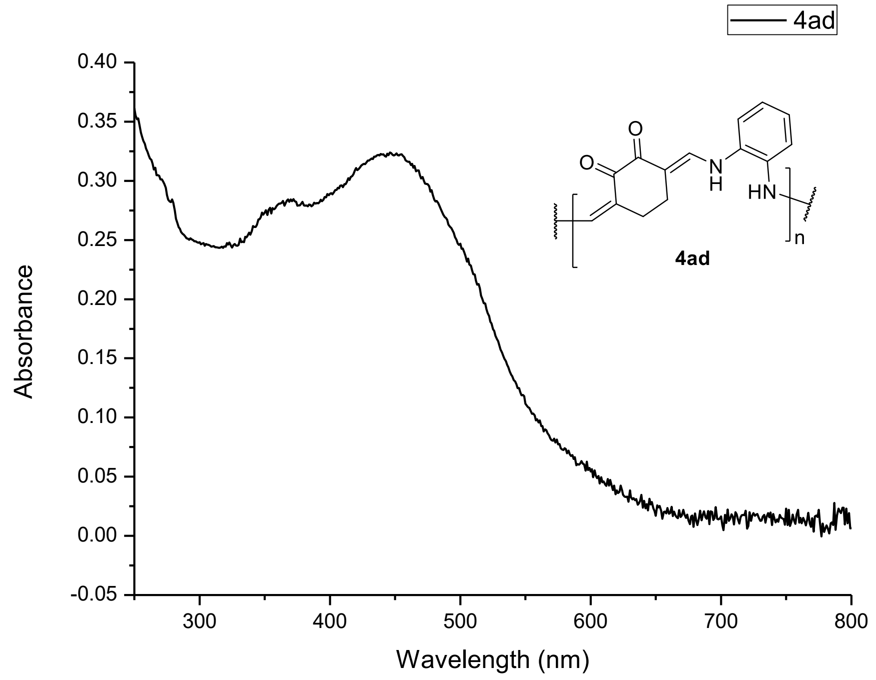
4ad
Absorbance
Wavelength (nm)
4ad
N
H
HN
n
O
O
0.40
0.35
0.30
0.25
0.20
0.15
0.10
0.05
0.00
-0.05
300
400
500
600
700
800

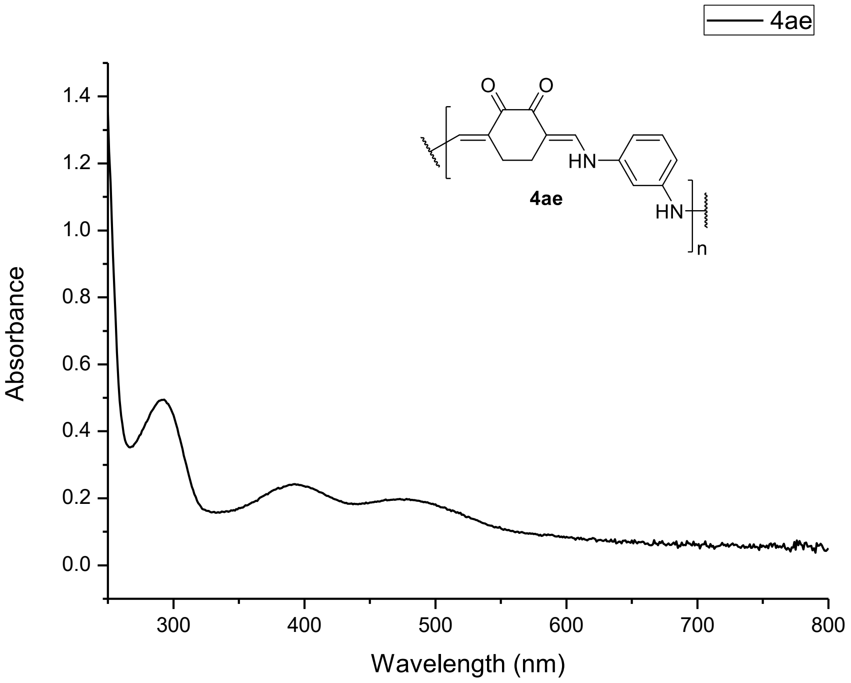
4ae
1.4
1.2
1.0
0.8
0.6
0.4
0.2
0.0
Absorbance
300
400
500
600
700
800
Wavelength (nm)
O
O
HN
HN
n
4ae

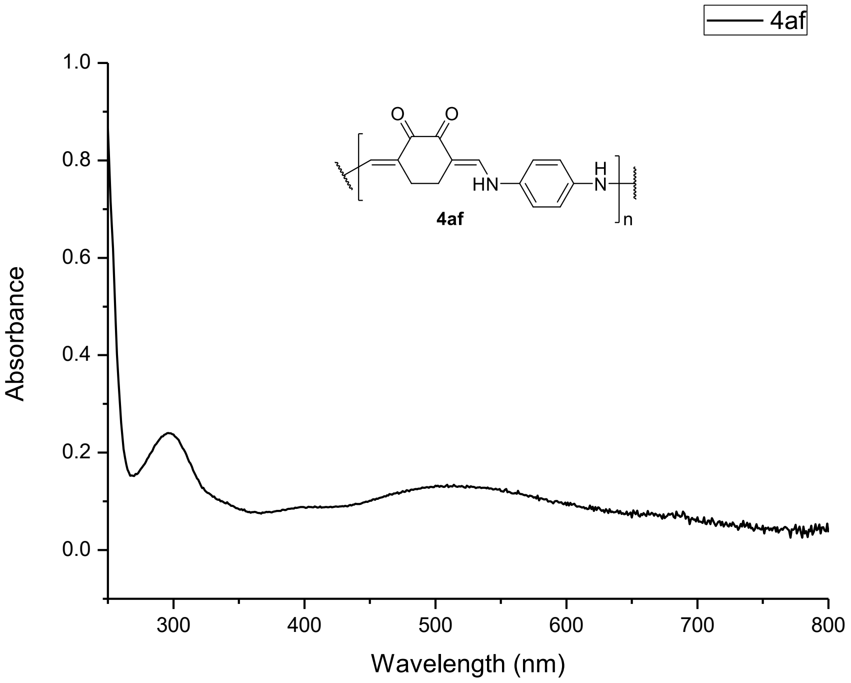
4af
1.0
0.8
0.6
0.4
0.2
0.0
Absorbance
300
400
500
600
700
800
Wavelength (nm)
O
O
HN
H
N
n
4af

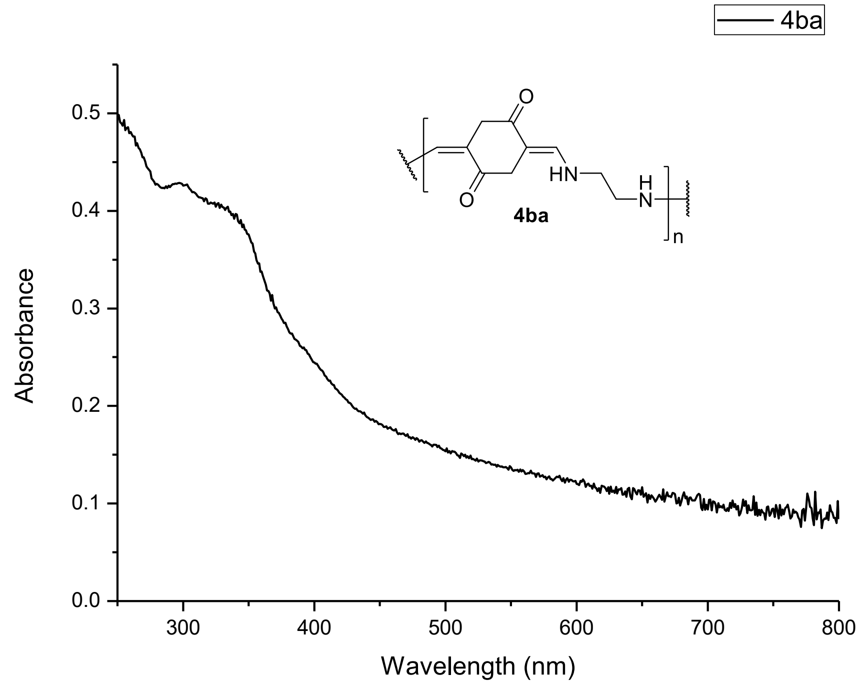
4ba
Absorbance
Wavelength (nm)
0.0
0.1
0.2
0.3
0.4
0.5
300
400
500
600
700
800
O
HN
H
N
n
4ba

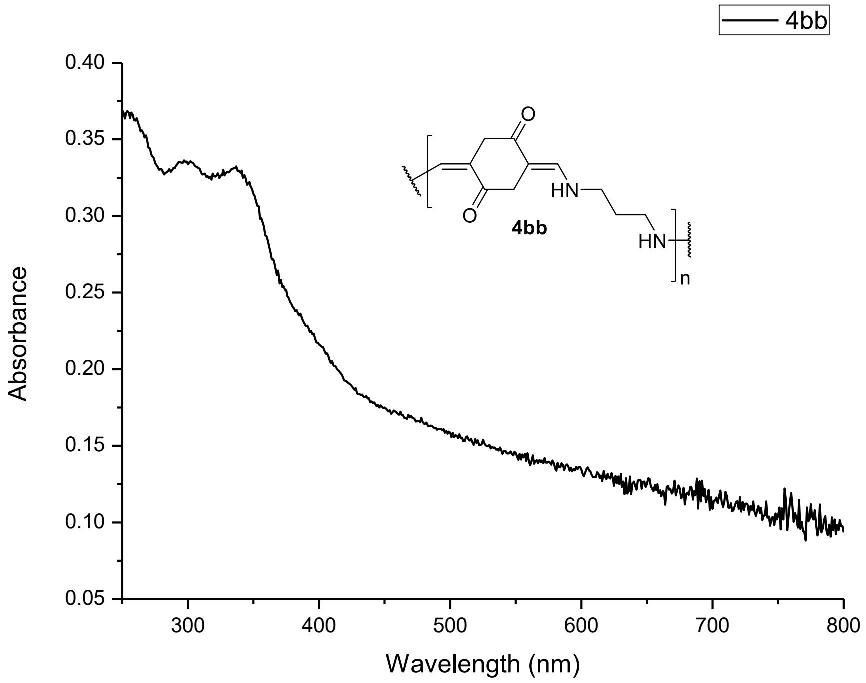
4bb
Absorbance
Wavelength (nm)
0.05
0.10
0.15
0.20
0.25
0.30
0.35
0.40
300
400
500
600
700
800
O
HN
n
4bb

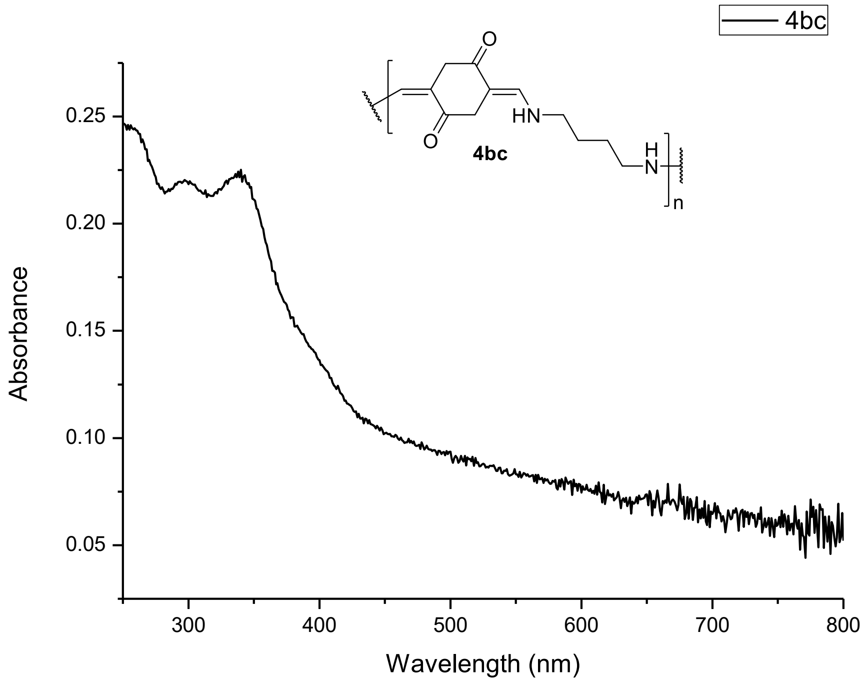
4bc
O
HN
O
4bc
H
N
n
Absorbance
0.25
0.20
0.15
0.10
0.05
300
400
500
600
700
800
Wavelength (nm)

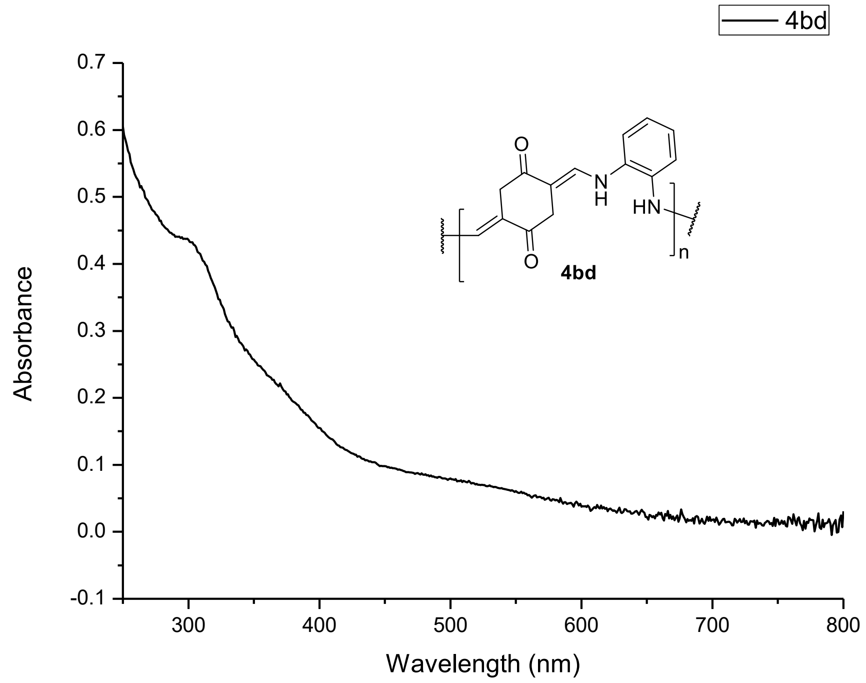
4bd
O
N
H
HN
O
4bd
n
Absorbance
0.7
0.6
0.5
0.4
0.3
0.2
0.1
0.0
-0.1
300
400
500
600
700
800
Wavelength (nm)

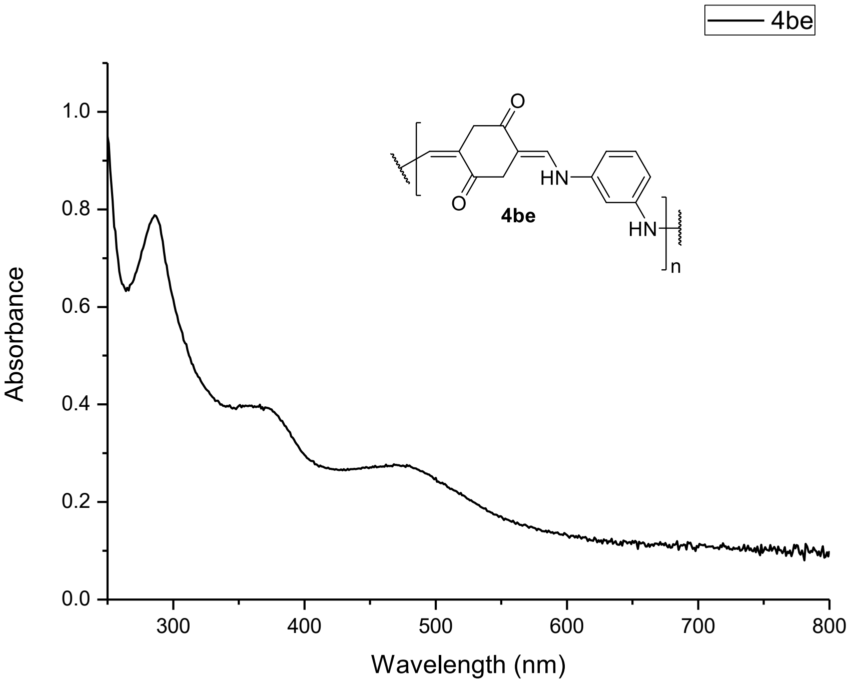
4be
1.0
0.8
0.6
0.4
0.2
0.0
Absorbance
300
400
500
600
700
800
Wavelength (nm)
O
HN
O
4be
HN
n

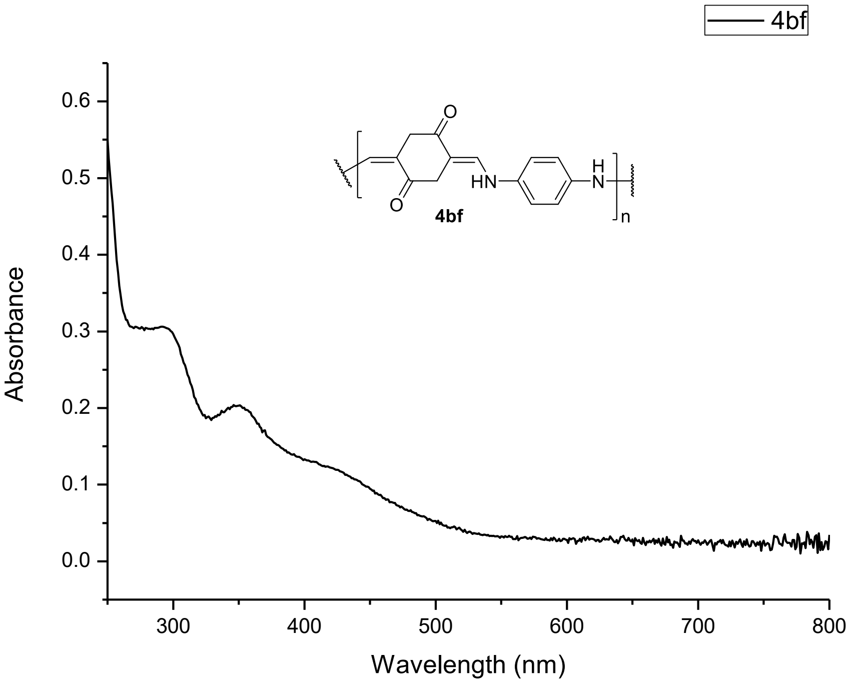
4bf
0.6
0.5
0.4
0.3
0.2
0.1
0.0
Absorbance
300
400
500
600
700
800
Wavelength (nm)
O
HN
H
N
O
4bf
n
